# HDGFRP3 interaction with 53BP1 promotes DNA double-strand break repair

**DOI:** 10.1093/nar/gkad073

**Published:** 2023-02-16

**Authors:** Zhen Zhang, William E Samsa, Yanyan De, Fan Zhang, Ofer Reizes, Alexandru Almasan, Zihua Gong

**Affiliations:** Department of Cancer Biology, Cleveland Clinic Lerner Research Institute, Cleveland, OH, USA; Department of Cancer Biology, Cleveland Clinic Lerner Research Institute, Cleveland, OH, USA; Department of Cancer Biology, Cleveland Clinic Lerner Research Institute, Cleveland, OH, USA; Department of Cancer Biology, Cleveland Clinic Lerner Research Institute, Cleveland, OH, USA; Department of Cardiovascular & Metabolic Sciences, Cleveland Clinic Lerner Research Institute, Cleveland, OH, USA; Department of Cancer Biology, Cleveland Clinic Lerner Research Institute, Cleveland, OH, USA; Department of Cancer Biology, Cleveland Clinic Lerner Research Institute, Cleveland, OH, USA

## Abstract

The 53BP1-dependent end-joining pathway plays a critical role in double-strand break (DSB) repair. However, the regulators of 53BP1 in chromatin remain incompletely characterized. In this study, we identified HDGFRP3 (hepatoma-derived growth factor related protein 3) as a 53BP1-interacting protein. The HDGFRP3–53BP1 interaction is mediated by the PWWP domain of HDGFRP3 and the Tudor domain of 53BP1. Importantly, we observed that the HDGFRP3–53BP1 complex co-localizes with 53BP1 or γH2AX at sites of DSB and participates in the response to DNA damage repair. Loss of HDGFRP3 impairs classical non-homologous end-joining repair (NHEJ), curtails the accumulation of 53BP1 at DSB sites, and enhances DNA end-resection. Moreover, the HDGFRP3–53BP1 interaction is required for cNHEJ repair, 53BP1 recruitment at DSB sites, and inhibition of DNA end resection. In addition, loss of HDGFRP3 renders BRCA1-deficient cells resistant to PARP inhibitors by facilitating end-resection in BRCA1 deficient cells. We also found that the interaction of HDGFRP3 with methylated H4K20 was dramatically decreased; in contrast, the 53BP1-methylated H4K20 interaction was increased after ionizing radiation, which is likely regulated by protein phosphorylation and dephosphorylation. Taken together, our data reveal a dynamic 53BP1-methylated H4K20–HDGFRP3 complex that regulates 53BP1 recruitment at DSB sites, providing new insights into our understanding of the regulation of 53BP1-mediated DNA repair pathway.

## INTRODUCTION

DNA double-strand breaks (DSBs) are highly toxic lesions that form when both strands of the DNA duplex are disrupted simultaneously. Generally, DSBs are repaired by two major pathways: non-homologous end joining (NHEJ) and homologous recombination (HR) (1). Studies show that BRCA1 or BRCA2 dysfunction profoundly sensitize cells to poly-ADP ribose polymerase (PARP) inhibitors (PARPi) due to HR repair defects in BRCA1 or BRCA2-deficient cancer cells (2–5). Unexpectedly, the loss of 53BP1 renders BRCA1-deficient cancer cells resistant to PARPi and ionizing radiation (IR) treatment (6,7), indicating that loss of 53BP1 expression is at least one determinant for how therapeutic resistance may arise in BRCA-mutated cancers.

53BP1 is an important mediator and effector of the DSB response (8). It contains 28 N-terminal SQ/TQ sites, a middle tandem Tudor domain, a ubiquitination-dependent recruitment (UDR) motif, and C-terminal BRCA1 carboxyl-terminal (BRCT) repeats (9). Recent studies demonstrated that 53BP1 is a key determinant of DSB repair pathway choice (6,7). Although 53BP1 has no apparent enzymatic activity, it contains interaction surfaces for numerous DSB-responsive proteins. We and others recently reported that mechanistically 53BP1 acts as an adaptor protein that controls two downstream sub-pathways: (i) one mediated by PTIP and Artemis (10,11); and (ii) the other mediated by RIF1 (12–16) and the downstream effectors MAD2L2 (17,18)/Shieldin complex (19–23), and the recently identified ASTE1 (24), to coordinate DSB repair pathway choices in BRCA1-deficient cells.

It is well known that the Tudor domain of 53BP1 interacts with H4K20me2, which is critical for 53BP1 recruitment to DSBs (25,26). Although the role of H4K20me2 in the recruitment of 53BP1 and the requirement of the 53BP1 tandem Tudor domain are well established (27,28), the underlying mechanism is still unclear. Even though nucleosomes are already highly dynamic structures that permit protein access even to buried sites of chromatin (29), the high abundance of H4K20me2 in cells indicates that this mark is usually not accessible to 53BP1 (30). One would assume that additional mechanisms must be responsible for exposure of H4K20me2. Indeed, Mallette *et al.* showed that RNF8-mediated degradation of JMJD2A is required to expose H4K20me2 for the recruitment of 53BP1 to DNA damage sites (31). In addition, RNF168 and RNF8 promote VCP (the AAA-ATPase valosin-containing protein)-mediated displacement of L3MBTL1 to allow efficient binding of 53BP1 to H4K20me2 (32,33). Conversely, we and others recently reported that TIRR (Tudor-Interacting Repair Regulator) associates with and stabilizes 53BP1 and prevents 53BP1 localization to sites of DNA damage by blocking its H4K20me2-binding sites (34–38).

In this study, we identified HDGFRP3 (hepatoma-derived growth factor-related protein 3) as a 53BP1-associated protein in the chromatin fraction. HDGFRP3 belongs to the hepatoma-derived growth factor (HDGF) related protein (HDGFR) family (39). All HDGFR family members have a highly conserved PWWP domain (40), which has been described as a potential histone methylation reader (41). It has been reported that HDGFRP3 is an endothelial growth factor, and activates the ERK pathway in endothelial cells (42), while the receptors on endothelial cells are yet to be validated. Moreover, HDGFRP3 increases the basal level of ERK phosphorylation/activation and enhances the duration of EGF-mediated activation of ERK1/2 in hepatocellular carcinomas cells (43), suggesting that HDGFRP3 may serve as a novel molecular target for treatment of hepatocellular carcinomas. Here, we investigated the functional significance of the HDGFRP3–53BP1 interaction in the DNA repair process.

## MATERIALS AND METHODS

### Cell culture and plasmids

HEK293T, MCF10A, MDA-MB-231, ES2, and U2OS cells were purchased from the American Type Culture Collection (ATCC) and cultured under conditions specified by the manufacturer. DR-GFP-U2OS, EJ5-GFP-U2OS and EJ7-GFP-U2OS cell lines were obtained from Jeremy Stark, Beckman Research Institute of the City of Hope, Duarte, CA. The DIvA cell line was obtained from Gaelle Legube, CNRS-University of Toulouse, France. The 53BP1 and HDGFRP3 cDNAs were subcloned into pDONR201 as an entry vector and subsequently transferred to gateway-compatible destination vectors for the expression of the triple-epitope tag SFB (S protein, FLAG, and streptavidin-binding peptide), HA, Myc, MBP and GST epitope fusion proteins. All deletion or point mutants were generated by site-directed mutagenesis and verified by DNA sequencing.

### Antibodies

The 53BP1 antibody we generated was described previously (11,15). The anti-FLAG M2 (#F3165), anti-RPA32 (#SAB1406400), anti-γH2AX (#05-636), anti-Histone H3 (#04-928), and anti-β-actin (#A5411) antibodies were purchased from MilliporeSigma. The anti-HDGFRP3 antibody was obtained from Assay Biotech (#C30644) and Proteintech (#12380-1-AP). The anti-α tubulin antibody (#2144S), anti-H2AX (#2595S), anti-GST antibody (#2625S), anti-H4K20me1 antibody (#9724SS), anti-H4K20me3 antibody (#5737S), anti-PP2A antibody sample kit (#9780T) and anti-53BP1 antibody (#4937S) were obtained from Cell Signaling Technology. The anti-CtIP antibody (#61141) was purchased from Active motif. The anti-GAPDH antibody (#sc-47724) and anti-53BP1 antibody (#sc-517281) were obtained from Santa Cruz. The anti-MBP antibody (#906901), anti-Rat 53BP1 antibody (#933002), anti-Rat Flag antibody (#637301), Alexa Fluor® 488 anti-PCNA (#307909), and Alexa Fluor® 488 anti-γH2AX (#613405) were purchased from BioLegend. The anti-HA antibody (#PI26183) was obtained from Thermo Fisher Scientific. The anti-H4K20me2 antibody was purchased from Diagenode (#C15200220).

### Immunofluorescence staining

Cells grown on coverslips were mock-treated or irradiated with a JL Shepherd Cs137 and allowed to recover for different time points. Cells were pre-extracted for 5 min with CSK buffer (100 mM NaCl, 300 mM sucrose, 10 mM Pipes, pH 6.8, 3 mM MgCl_2_) with 0.5% Triton X-100, and then fixed in 4% paraformaldehyde solution for 10 min. Cells were incubated with primary antibodies diluted in 5% goat serum at room temperature for 2 h. Coverslips were washed and incubated with secondary antibodies for 1 h at room temperature. Cells were then stained with DAPI to visualize nuclear DNA. The coverslips were mounted onto glass slides with anti-fade solution and visualized using the Nikon Eclipse E800 fluorescence microscope.

### Co-immunoprecipitation and western blotting

Cells were lysed with NTEN buffer (20 mM Tris–HCl, pH 8.0, 100 mM NaCl, 1 mM EDTA, 0.5% Nonidet P-40) containing protease inhibitors, phosphatase inhibitors, 2 mM MgCl_2_ and Benzonase at 4°C for 30 min. Cleared cell lysates were incubated with either Protein A agarose bead coupled with anti-HDGFRRP3 antibody, anti-53BP1 antibody, or streptavidin sepharose beads for 3 h at 4°C. Beads were then washed and boiled in 2x Laemmli buffer and separated by SDS-PAGE. PVDF membranes were blocked in 5% milk in TBST buffer and then probed with the indicated antibodies.

### Isolation of chromatin fraction

Cells were washed twice with PBS before resuspending in NTEN buffer supplemented with protease inhibitors and incubated on ice for 30 min. By centrifuging at 8,000 rpm for 5 min at 4°C, nuclei were isolated, washed four times with low salt buffer (10 mM Tris, pH 7.4, 0.2 mM MgCl_2_) and subsequently lysed in chromatin extraction buffer (50 mM Tris, pH 8.0, 300 mM NaCl, 1% NP40, 1 mM DTT) with protease inhibitors, and the cell suspension was sonicated on ice. After ultracentrifugation, the supernatant was transferred to a new tube as the chromatin fraction.

### Recombinant protein production

The GST proteins expressed in *Escherichia coli* were purified by Glutathione Sepharose 4B beads and eluted by glutathione buffer (20 mM l-gluthathione, 50 mM Tris–HCl, pH 9.0). The MBP proteins expressed in *E. coli* were purified by amylose resin and eluted by maltose buffer (10 mM maltose; 20 mM Tris–HCl, pH 7.4; 200 mM NaCl; 1 mM EDTA; 10 mM β-mercaptoethanol).

### Pull-down assays using bacterially expressed fusion proteins

GST or MBP fusion proteins were expressed in *E. coli* and purified. GST-HDGFRP3 protein (10 μg) was immobilized on 20 μl glutathione Sepharose 4B beads in 500 μl NTEN buffer containing protease inhibitors, 2 mM MgCl_2_ and 500 U/ml Benzonase for 1 h at 4°C. The beads were collected by centrifugation and washed 3 times with NTEN buffer. MBP-53BP1 Tudor domain protein (10 μg) or lysates prepared from cells transiently transfected with plasmids encoding the indicated proteins in NTEN buffer containing 2 mM MgCl_2_ and 500 U/ml Benzonase were added and incubated with beads for 3 h at 4°C. The supernatant was removed, and the beads were washed 6 times with NTEN buffer. The resulting samples were subjected to SDS-PAGE and analyzed by western blotting.

### Peptide pull-down assays

The biotinylated histone H4K20me peptide was incubated with bacterially expressed and purified protein of MBP-53BP1 Tudor domain, GST-HDGFRP3 or MBP-HDGFRP3 PWWP in TNB buffer [50 mM Tris–HCl (pH 8.0), 150 mM NaCl, 0.05% NP40, 0.1% BSA]. After 4 h at 4°C, streptavidin beads were added, and incubation was continued for 30 min at 4°C. Beads were washed 6 times with TNB buffer, and boiled in 2x Laemmli buffer, and proteins were separated by SDS-PAGE.

### *In situ* proximity ligation assay (PLA)

U2OS cells were cultured on coverslips, fixed with 4% paraformaldehyde for 10 min and permeabilized with 0.5% Triton X-100 for 5 min, followed by 1 h in blocking buffer. For the visualization of protein interactions, samples were incubated with the primary antibodies for 1 h at room temperature. *In situ* PLA was performed according to the manufacturer's protocol (Duolink® Proximity Ligation Assay, Sigma) using PLA probe anti-mouse MINUS and PLA probe anti-rabbit PLUS.

In order to visualize the protein co-localization at DSB sites, a combined PLA and immunofluorescence was performed. Briefly, the slides were incubated with an anti-rat 53BP1 antibody concomitantly with anti-rabbit HDGFRP3 and anti-mouse 53BP1 antibodies (for PLA). After washing, the slides were then incubated with an appropriate secondary antibody, chicken anti-rat-Alexa Fluor-488, concurrently with the PLA probes. Due to lack of host species for γH2AX other than rabbit and mouse, the Alexa-Fluor 488 (AF488) mouse γH2AX was used in the reaction to directly visualize the γH2AX foci coupled with PLA signals.

### CRISPR–cas9 gene-editing approach to generate HDGFRP3 knockout cells

Sequence designs were chosen to target the HDGFRP3 gene in the first few exons and were tested for obvious potential off-targets by bioinformatics analysis. Plasmids expressing short-guide RNAs (sgRNAs) were constructed by inserting a pair of annealed oligonucleotides encoding the corresponding sgRNA into the PX330-mCherry vector (Addgene). MCF10A, MDA-MB-231 or ES2 cells were transfected with PX330-based plasmid vectors expressing HDGFRP3 sgRNA, the Cas9 nuclease, and the mCherry protein. Cells were then sorted and seeded as single colonies in 96-well plates by fluorescence-activated cell sorting (FACS). After 2 weeks, clones were selected based on Western blotting with the HDGFRP3 antibody. In addition, genomic DNA was extracted from cell lines arising from single clones. PCR reactions to amplify targeted loci were performed, and agarose gel electrophoresis was used to confirm the correct size of PCR products. PCR products were then cloned into the pCR2.1-TOPO vector and transformed into DH5α competent cells. Plasmid DNA was isolated from multiple colonies obtained from each transformation and sequenced to ensure frameshift mutations in the targeted region.

The sequence information for sgRNAs used for HDGFRP3 knockout cell generation is as follows:

- HDGFRP3_sgRNA1: GCGGCCCCGCGAGTACAAAG;
- HDGFRP3_sgRNA2: GAAGGGCTACCCGCACTGGC;

### siRNA or sgRNA-mediated knockdown

siRNAs targeting HDGFRP3 were transfected to DR-GFP-U2OS, EJ5-GFP-U2OS or EJ7-GFP-U2OS cells using Lipofectamine 3000 (Life Technologies) according to the manufacturer's instructions. For the PARPi resistance assay, pLentiCRISPR v2 vector-based sgRNA constructs together with packaging vectors psPAX2 and pMD2.G were used for lentiviral production. The indicated cells were infected with lentivirus for 48 h and then selected with medium containing puromycin (2 μg/ml). The knockdown efficiency was confirmed by western blot analysis.

- HDGFRP3_siRNA1: GAUUGUGGGAAAUAGAAAA;
- HDGFRP3_siRNA2: CUGCAUUUCUAGGUCCCAA.

### DSB repair assays

The well-established DR-GFP U2OS and EJ5-GFP-U2OS reporter cell lines (44,45) were used to measure HR and NHEJ, respectively. In brief, both reporter cell lines were transfected with siRNAs for control or HDGFRP3 for 24 h, then transfected with pCBASceI vector and pcDNA3.1-mCherry at 9:1 ratio for another 48 h. The GFP+ and mCherry+ cell population was quantitated and the NHEJ or HR repair rate represented as GFP+/mCherry+.

### Classical NHEJ (c-NHEJ) repair assay

EJ7-GFP U2OS reporter cells were developed to detect distal end joining without indels on DSB ends (46). The GFP coding sequence at the GGC codon was inserted as a 46-nucleotide spacer between the GG bases and the C base. Two sgRNAs, 7a and 7b, target Cas9-induced DSBs to excise the 46-nt spacer. In brief, EJ7-GFP U2OS reporter cells were transfected with siRNAs for control or HDGFRP3 for 24 h, then transfected with mCherry and two sgRNAs, 7a and 7b at 1:9 ratio for another 48 h. The GFP+ and mCherry+ cell population was quantitated and the cNHEJ repair rate represented as GFP+/mCherry+.

### Random plasmid integration assay

Assays were performed as previously described (47) with minor modifications. Briefly, MDA-MB-231 and its derivative HDGFRP3 KO cells were transfected with BamHI-XhoI linearized pEGFP-C1 (Clontech). The following day, transfected cells were trypsinized and seeded in 10 cm dishes at different densities for colony formation. Selection was initiated the following day with 0.5 mg/ml G418. The cells on a plate lacking G418 were fixed to assess transfection efficiency. Colonies were then stained and counted. Random-plasmid integration events were normalized to transfection and plating efficiencies.

### Clonogenic survival assays

An ionizing radiation (IR) sensitivity assay was carried out as described previously (48). Briefly, a total of 800 cells were seeded onto 60-mm dishes in triplicate and treated with IR or DNA damaging agents the next day. Cells were then incubated for 14 days. Resulting colonies were fixed and stained with Coomassie Blue. Colony numbers were counted, and results were summarized as the mean of data obtained from three independent experiments.

### DNA end resection assay

Briefly, DIvA cells were treated with 600 nM 4-Hydroxytamoxifen (4-OHT) for 4 h or mock-treated. Genomic DNA was extracted. The genomic DNA sample (∼400 ng) was subjected to an RNase H treatment for 15 min before mock digestion or digestion with the restriction enzyme *BsrGI* (DSB, chromosome 1) or *HindIII* (No DSB, chromosome 22) at 37°C overnight. Samples were heat-inactivated at 65°C for 10 min and were used as templates for qPCR. To quantify the extent of resection, the digested or mock-digested samples were amplified by qPCR using primers that are described in Supplemental Table S1. The percentage of ssDNA (ssDNA %) generated by resection at selected sites was calculated based on the following equation: ssDNA % = 1/(2^(△Ct – 1)^ + 0.5) × 100. △Ct was calculated by subtracting the Ct value of the mock-digested sample from the Ct value of the digested sample. At least three biological repeats were performed.

### Cell cycle analysis

The indicated cells were collected and fixed with 75% (vol/vol) ethanol at -20°C overnight and then resuspended in PBS containing RNase A (100 μg/ml) at 37°C for 15 min. 200–400 μl of PI (final concentration 50 μg/ml) was added to the cells. Cell cycle distribution was analyzed by flow cytometry. Data were collected from 25,000 events per sample.

### Tandem affinity purification

293T cells were transfected with plasmids encoding the SFB-53BP1 IRIF (ionizing radiation-induced foci) region or SFB-HDGFRP3. Cell lines stably expressing tagged proteins were selected, and the expression of exogenous proteins was confirmed by immunoblotting and immunostaining. For tandem affinity purification, 293T cells stably expressing the SFB-53BP1 IRIF region or SFB-HDGFRP3 were collected and lysed with NTEN buffer on ice for 20 min. Crude lysates were removed by centrifugation, and the pellets were suspended in nuclease buffer (10 mM HEPES, pH 7.4, 10 mM KCl, 0.5 mM MgCl_2_, 2 mM CaCl_2_ and 1 μg/ml of each of pepstatin A and aprotinin) supplemented with 150 U/ml micrococcal nuclease S7 and incubated in a 37°C water bath for 5 min until the suspension turned cloudy. Then the chromatin fraction was collected by centrifugation, and the supernatants were incubated with streptavidin-conjugated beads for 3 h at 4°C. The immunocomplexes were washed three times with the NTEN buffer and then bead-bound proteins were eluted twice with NTEN buffer containing 1 mg/ml biotin. The eluates were incubated with S protein beads. The immunocomplexes were again washed three times with NTEN buffer and subjected to SDS-PAGE. Protein bands were excised, digested and the peptides were analyzed by mass spectrometry (performed by the Taplin Biological Mass Spectrometry Facility, Harvard University).

### Statistical analysis

The reported values are the mean and S.E. of three independent experiments. Statistical analysis was performed using Student's *t*-test and one-way ANOVA test. *P* < 0.05 was considered as statistically significant.

## RESULTS

### HDGFRP3 is a novel 53BP1-associated protein

**Figure 1.**
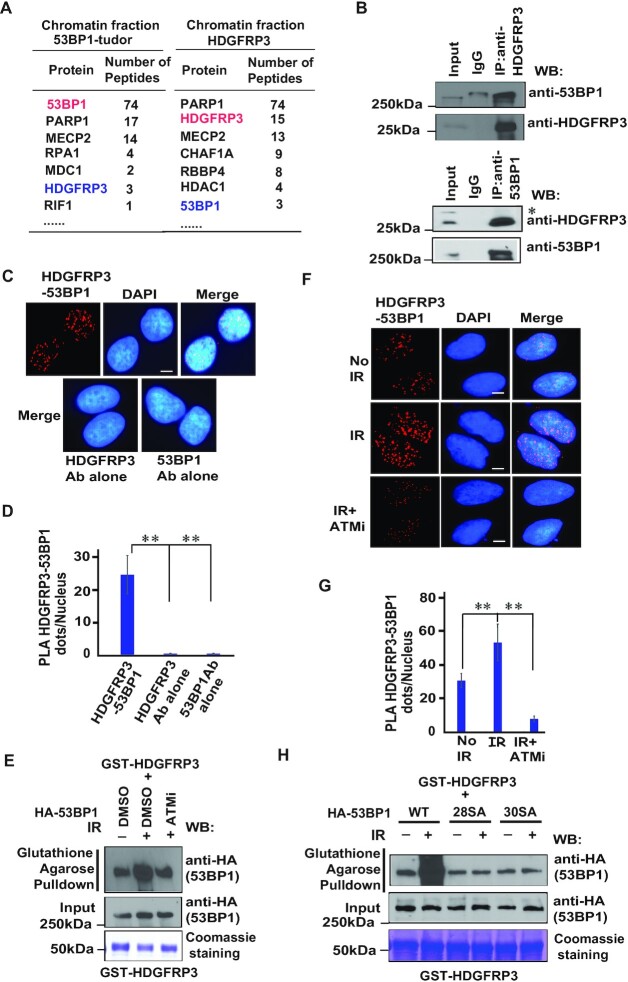
HDGFRP3 is a novel 53BP1-associated protein. (**A**) A List of 53BP1 IRIF/HDGFRP3- associated proteins identified in the chromatin fractions by mass spectrometric analysis. (**B**) Endogenous HDGFRP3 interacts with 53BP1. 293T cell lysates were prepared and immunoprecipitated with HDGFRP3 (Proteintech, upper panel) or 53BP1 (Santa Cruz, lower panel) antibodies followed by immunoblotting with the 53BP1 (Cell Signaling Technology) and HDGFRP3 (Proteintech) antibodies. The bands marked by an asterisk represent non-specific bands. (**C**) PLA detection of the HDGFRP3–53BP1 interaction. U2OS cells were subjected to PLA using the 53BP1 (MilliporeSigma) and HDGFRP3 (Proteintech) antibodies, shown as distinct fluorescent dots (upper panel). Negative controls used one of these two antibodies. Scale bar, 10 μm. (**D**) Quantification of the results in (C). PLA dots per nucleus were quantified in 100 cells for each experiment (lower panel). Data are represented as the mean ± S.E. (*n* = 3). **P < 0.01. (**E**) The 53BP1–HDGFRP3 interaction is regulated by DNA damage and ATM by GST pulldown assay. HEK293T cells with expressed HA-53BP1 were either left untreated, irradiated with 10 Gy, or treated with ATMi (KU-60019) for 1 h prior to irradiation with 10 Gy. One hour later, beads coated with bacterially expressed GST-HDGFRP3 fusion protein were incubated, respectively, with cell lysates containing exogenously expressed HA-53BP1. Immunoblotting experiments were carried out using the indicated antibodies. Similar results were obtained from three biologically independent experiments. (**F**) The 53BP1–HDGFRP3 interaction is dependent of both DNA damage and ATM as determined by the PLA assay. U2OS cells were either left untreated, irradiated with 10 Gy or treated with ATMi (KU-60019) for 1 h prior to irradiation with 10 Gy. One hour later, a PLA assay was performed using the 53BP1 (MilliporeSigma) and HDGFRP3 (Proteintech) antibodies, shown as distinct fluorescent dots (left panel). Scale bar, 10 μm. (**G**) Quantification of the results in (F). PLA dots per nucleus were quantified in 100 cells for each experiment (right panel). Data are represented as the mean ± S.E. (*n* = 3). **P < 0.01. (**H**) The 53BP1–HDGFRP3 interaction is regulated by ATM-mediated 53BP1 phosphorylation. HEK293T cells expressing wild-type (WT), 28SA or 30SA mutants of HA-53BP1 were either left untreated or irradiated with 10 Gy. One hour later, beads coated with bacterially expressed GST-HDGFRP3 fusion protein were incubated, respectively, with cell lysates containing exogenously expressed WT, 28SA or 30SA mutants of HA-53BP1. Immunoblotting experiments were carried out using the indicated antibodies. Three biologically independent experiments were performed, with similar results obtained.

The ionizing radiation (IR)-induced Foci (IRIF) region of 53BP1 is composed of the Tudor domain and the ubiquitination-dependent recruitment (UDR) motif, which are required for the accumulation of 53BP1 at DNA damage sites (49,50). To understand how 53BP1 is regulated at DNA break sites after DNA damage, we performed tandem affinity purification (TAP) using chromatin fractions derived from HEK293T cells stably expressing the SFB (S protein, FLAG, and streptavidin-binding peptide)-tagged IRIF region of 53BP1. Mass spectrometry analysis revealed that HDGFRP3 is a novel 53BP1-associated protein (Figure 1A). HDGFRP3 is a 203 amino acids protein containing a PWWP domain with unknown function in DNA damage response. Furthermore, we repeatedly identified 53BP1 as a HDGFRP3-associated protein through TAP, using lysates derived from HEK293T cells that stably expressing SFB-tagged human HDGFRP3 (Figure 1A). In addition, we confirmed that the HDGFRP3–53BP1 interaction occurs between endogenous proteins (Figure 1B) with immunoprecipitation of the specific HDGFRP3 antibody (Supplemental Figure S1A) and the 53BP1 antibody, respectively, suggesting that these two proteins indeed associate with each other *in vivo*.

The *in situ* proximity ligation assay (PLA) allows direct visualization, as well as quantification of *in situ* interactions (two proteins that are in close vicinity) in fixed cells. PLA detection of the HDGFRP3–53BP1 interaction was visualized as distinct fluorescent dots in U2OS cells (Figure 1C, D). This interaction was specific, since only very few PLA signals were observed when the HDGFRP3 and 53BP1 antibodies were used alone or when 53BP1 was depleted (Figure 1C, D, Supplemental Figure S1B–D). To further characterize the interaction between HDGFRP3 and 53BP1, we investigated other conditions that might regulate this interaction. We performed the PLA assay in U2OS cells subjected to IR, found that the HDGFRP3–53BP1 interaction was enhanced following IR (Supplemental Figure S1E-S1F). We also performed TAP using damaged chromatin fraction. Mass spectrometry analysis revealed an increased number of peptides for HDGFRP3 and 53BP1 in a reciprocal purification (Supplemental Figure S1G), further confirming that the HDGFRP3–53BP1 interaction is enhanced following DNA damage. However, the HDGFRP3–53BP1 interaction was not affected when the extracts were treated with the Benzonase nuclease, suggesting that the interaction was not mediated by DNA (Supplemental Figures S1H, S12A).

We next investigated how the HDGFRP3–53BP1 interaction may be regulated after DNA damage. In this regard, a GST pull-down assay was performed in the HEK293T cells expressing HA-53BP1 either left untreated, treated with IR or with ATMi prior to IR. We found that the interaction of GST-HDGFRP3 with HA-53BP1 was induced by DNA damage, and it was mainly dependent on ATM kinase (Figure 1E, Supplemental Figure S11A). In agreement with the pulldown experiment, this IR-induced increased interaction is prevented if cells were treated with the ATM inhibitor (ATMi) as determined by the PLA assay (Figure 1F, G). 53BP1 undergoes ATM-dependent phosphorylation at 28 S/T-Q sites at its N terminus, which is important to regulate the DSB repair function of 53BP1 (10). In addition, S1778-53BP1 is known to be a substrate of ATM and DNA-PKcs (51,52) and S1317-53BP1 is phosphorylated by AMPK (53), which are required to promote 53BP1 recruitment and NHEJ repair. A GST pulldown experiment was performed (Figure 1H, Supplemental Figure S11B). Comparing the increased interaction of HDGFRP3 with wild-type (WT) 53BP1, the interaction of HDGFRP3 with a mutant with all these 28 S/T phosphorylation sites mutated to A (designated as the 28SA mutant), or a mutant containing S1317AS1778A double mutant in addition 28SA mutant (designated as the 30SA mutant) of 53BP1 is decreased following IR. Moreover, the 30SA mutant of 53BP1 did not further decrease its binding to HDGFRP3 after IR, indicating that the two phosphorylation residues (S1317, S1778) are not important for the HDGFRP3–53BP1 interaction following DNA damage. Taken together, these data indicate that DNA damage-induced and ATM-dependent phosphorylation of 53BP1 likely control the 53BP1–HDGFRP3 interaction.

### The HDGFRP3–53BP1 interaction is mediated by the PWWP domain of HDGFRP3 and the tudor domain of 53BP1

**Figure 2.**
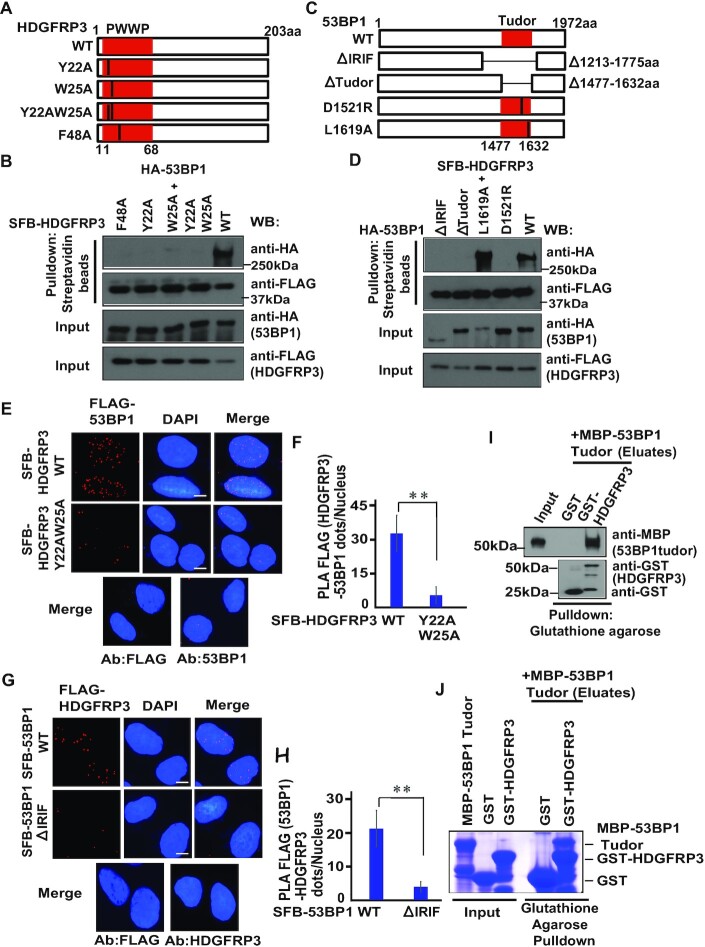
The HDGFRP3–53BP1 interaction is mediated by the PWWP domain of HDGFRP3 and the Tudor domain of 53BP1. (**A**) Schematic representation of the WT and mutants of HDGFRP3 used in this study. (**B**) The PWWP domain of HDGFRP3 is required for the HDGFRP3–53BP1 interaction. HEK 293T cells were transfected with plasmids encoding HA-tagged 53BP1 along with plasmids encoding the WT or mutants of SFB-tagged HDGFRP3. Immunoprecipitation (IP) reactions were conducted with streptavidin beads and subjected to western blotting with the indicated antibodies. (**C**) Schematic representation of the WT and mutants of 53BP1 used in this study. (**D**) The Tudor domain of 53BP1 is responsible for its interaction with HDGFRP3. HEK293T cells were transfected with plasmids encoding SFB-tagged HDGFRP3 along with plasmids encoding the WT or mutants of HA-tagged 53BP1. IP reactions were conducted with streptavidin beads and subjected to western blotting with the indicated antibodies. (**E**) PLA detection of the interaction between SFB-HDGFRP3 and endogenous 53BP1. U2OS cells were transfected with SFB-HDGFRP3 WT and the Y22AW25A mutant. The PLA assay was performed using both the 53BP1 (11,15) and FLAG (MilliporeSigma) antibodies, shown as distinct fluorescent dots. Negative controls used either one of the two antibodies. Scale bar, 10 μm. (**F**) Quantification of the results in (E). PLA dots per nucleus were quantified in 100 cells for each experiment. Data are represented as the mean ± S.E. (*n* = 3). **P < 0.01. (**G**) PLA detection of the interaction between SFB-53BP1 and endogenous HDGFRP3. U2OS cells were transfected with SFB-53BP1 WT and the ΔIRIF mutant. The PLA assay was performed using the HDGFRP3 (Proteintech) and FLAG (MilliporeSigma) antibodies, shown as distinct fluorescent dots. Negative controls used either one of the two antibodies. Scale bar, 10 μm. (**H**) Quantification of the results in (G). PLA dots per nucleus were quantified in 100 cells for each experiment. Data are represented as the mean ± S.E. (*n* = 3). **P < 0.01. (**I**) Analysis of the interaction between HDGFRP3 and the 53BP1 tudor domain using a GST pull-down assay. Bacterial purified MBP-53BP1 tudor domain protein was mixed with bacterial purified GST or GST–HDGFRP3 protein. The bound proteins were pulled down using Glutathione Sepharose 4B. The resulting samples were subjected to SDS–PAGE. Blots were probed with the indicated antibodies. Three biologically independent experiments were performed, with similar results obtained. (**J**) Analysis of the interaction between HDGFRP3 and the 53BP1 Tudor domain using a GST pull-down assay. Bacterial purified MBP-53BP1 Tudor protein was mixed with bacterial purified GST or GST–HDGFRP3 protein. The bound proteins were pulled down using Glutathione Sepharose 4B and eluted before analysis using SDS–PAGE. The gel was stained with Coomassie blue. Three biologically independent experiments were performed, with similar results obtained.

HDGFRP3 belongs to the HDGFR family, which has a commonly conserved N-terminal PWWP domain (Figure 2A). The PWWP domain contains several highly conserved amino acids and mainly mediates protein-protein interactions. Thus, we constructed several mutations in the PWWP domain of HDGFRP3 and performed co-immunoprecipitation (Co-IP) experiments. As shown in Figure 2B, none of these HDGFRP3 mutants interact with 53BP1, demonstrating that the PWWP domain of HDGFRP3 is critical for its binding to 53BP1. Next, we sought to define the HDGFRP3 binding region on 53BP1, so we generated a series of 53BP1 mutants (Figure 2C). Of note, previous studies have demonstrated that the D1521R mutant disrupts the function of the Tudor domain, and the L1619A mutation abolishes the function of the UDR motif (50,54), By performing a Co-IP experiment, we observed that deletions of either the IRIF region or the Tudor domain of 53BP1 led to a dramatic decrease in the 53BP1–HDGFRP3 interaction (Figure 2D). Moreover, mutation of the D1521 residue within the Tudor domain disrupted the 53BP1–HDGFRP3 interaction, whereas the L1619A mutation within the UDR motif had no effect on the 53BP1–HDGFRP3 interaction (Figure 2D, Supplemental Figure S2A, B). These findings indicate that the Tudor domain of 53BP1 is responsible for the 53BP1–HDGFRP3 interaction.

To further confirm the specificity of the HDGFRP3–53BP1 interaction, the PLA assay was performed in U2OS cells. In agreement with the Co-IP, the HDGFRP3–53BP1 interaction was also detected *in situ* by PLA (Figure 2E–H). Moreover, a significantly lower number of PLA dots per nucleus was observed with the Y22AW25A mutant of HDGFRP3, compared to cells transfected with WT HDGFRP3 (Figure 2E, F, Supplemental Figure S2C). In addition, cells transfected with a 53BP1 fragment (ΔIRIF) displayed much fewer dots per nucleus (Figure 2G-H, Supplemental Figure S2D), showing the specific involvement of the IRIF domain in the interaction of HDGFRP3 and 53BP1. No difference in transfection efficiency or immunofluorescence intensity of these transfected plasmids was observed (Supplemental Figure S2E, F). Taken together, these data suggest that HDGFRP3 interacts with 53BP1, and that the HDGFRP3–53BP1 interaction is mediated by the PWWP domain of HDGFRP3 and the Tudor domain of 53BP1. To confirm that this interaction was indeed direct, a GST pulldown approach was used. Using a bacterially expressed and purified GST-fused HDGFPR3 protein and MBP-fused 53BP1 Tudor domain protein, we show that the GST-HDGFRP3 binds to the MBP-53BP1 Tudor domain *in vitro* (Figure 2I, J), indicating that HDGFRP3 directly binds to the 53BP1 Tudor domain.

### HDGFRP3 is recruited to DSB sites

To visualize whether HDGFRP3 is present at sites of DNA damage, we performed the PLA assay in fixed cells after irradiation. As shown in Figure 1F, G, Supplemental Figure S1E, F, the PLA assay revealed accumulation of HDGFRP3 at DNA damage sites through its association with 53BP1 in response to irradiation.

**Figure 3.**
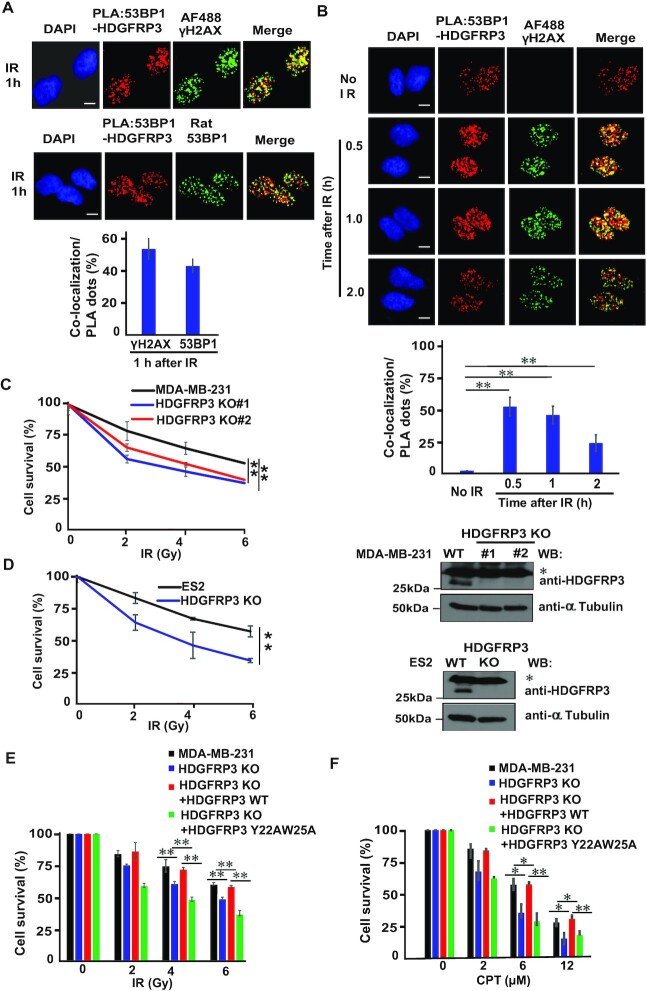
HDGFRP3 is recruited to DNA damage sites. (**A**) The 53BP1–HDGFRP3 complex co-localizes with 53BP1 and γH2AX at DSBs. U2OS cells were irradiated with 10 Gy and processed 1 h later for a combined application of immunofluorescence and *in situ PLA* as described in the ‘Materials and methods.’ PLA signals were recognized as red fluorescent spots. The co-localization between PLA signal and 53BP1 or γH2AX revealed by the yellow pixels. Scale bar, 10 μm. Quantification of the results in (A). The percentage of co-localization per PLA dots were quantified in 100 cells for each experiment (lower panel). Data are represented as the mean ± S.E. (n = 3). (**B**) The 53BP1–HDGFRP3 complex co-localizes with γH2AX at DSBs. U2OS cells were either left untreated or irradiated with 10 Gy, followed by recovery at the indicated time points. A combined application of immunofluorescence and *in situ PLA* was performed as described in the ‘*Materials and methods*.’ PLA signals were recognized as red fluorescent spots. The co-localization between PLA signal and γH2AX revealed by the yellow pixels (upper panel). Scale bar, 10 μm. Quantification of the results in (B). The percentage of co-localization per PLA dots were quantified in 100 cells for each experiment (lower panel). Data are represented as the mean ± S.E. (*n* = 3). **P < 0.01. (**C**) HDGFRP3 loss increases cellular sensitivity to IR. The MDA-MB-231 breast cancer cell line or its derivative HDGFRP3 knockout (KO) cells were mock-treated or treated with different doses of IR. Colony formation was quantified relative to colonies formed in untreated cells from the same setting (left panel). Data are represented as the mean ± S.E. (*n* = 3). **P < 0.01. Western blotting analysis was performed to verify the KO of HDGFRP3 (right panel). The bands marked by an asterisk represent non-specific bands. (**D**) HDGFRP3 loss increases cellular sensitivity to IR. The ES2 ovarian cancer cell line or its derivative HDGFRP3 KO cells were mock-treated or treated with different doses of IR and cell survival determined by the clonogenic assay (left panel). Data are represented as the mean ± S.E. (*n* = 3). **P < 0.01. Western blotting analysis was performed to verify the KO of HDGFRP3 (right panel). The bands marked by an asterisk represent non-specific bands. (E and F) A defect in interaction between 53BP1 and HDGFRP3 increases cellular sensitivity to DNA damage. HDGFRP3 knock-out MDA-MB-231 cells were reconstituted with sgRNA-resistant WT or Y22AW25A of SFB-tagged HDGFRP3. These cells were mock-treated or treated with different doses of IR (**E**) or Camptothecin (CPT) (**F**). Colony formation was quantified relative to colonies formed in untreated cells from the same setting. Data are represented as the mean ± S.E. (*n* = 3). *P < 0.05; **P < 0.01.

We failed to observe HDGFRP3 foci formation after IR using the HDGFRP3 antibody we generated and the commercial antibodies (data not shown). Thus, we applied a combined immunofluorescence and *in situ* PLA to investigate the co-localization of PLA signals (HDGFRP3–53BP1 interaction) with the DSB marker γH2AX or 53BP1 at sites of DSB. As shown in Figure 3A, we indeed observed co-localization of PLA signals with γH2AX or 53BP1 at sites of DSB following IR. To further characterize the kinetics of co-localization of γH2AX with the HDGFRP3–53BP1 interaction, we performed the combined immunofluorescence–PLA approach in U2OS cells subjected to IR followed by recovery at the indicated time points. We found that the co-localization of γH2AX with the HDGFRP3–53BP1 interaction was enhanced at the early time-point (0.5 and 1 h) following IR (Figure 3B), indicating kinetic changes of the HDGFRP3–53BP1 interaction at the site of DSB following DNA damage.

Because HDGFRP3 is recruited to DSB sites, we hypothesized that HDGFRP3 loss would lead to IR sensitivity. To this end, we generated HDGFRP3 knockout (KO) cells by CRISPR/Cas9 in MDA-MB-231 breast cancer (Figure 3C) and ES2 ovarian cancer cells (Figure 3D). Indeed, cell survival following the IR treatment of the MDA-MB-231 and ES2 cell lines with KO of HDGFPR3 demonstrated that loss of HDGFRP3 resulted in increased sensitivity to IR (Figure 3C, D). Moreover, reconstitution of WT HDGFRP3 decreased cell sensitivity to IR, however reconstitution with the Y22AW25A mutant of HDGFRP3 failed to do so (Figure 3E). Similar results were obtained from cells treated with a topoisomerase inhibitor Camptothecin (CPT) (Figure 3F). These data indicated that the PWWP domain of HDGFRP3, which is required for its interaction with 53BP1, is important for the function of HDGFRP3 in response to DNA damage and its DNA repair.

### HDGFRP3 participates in DNA damage and repair

**Figure 4.**
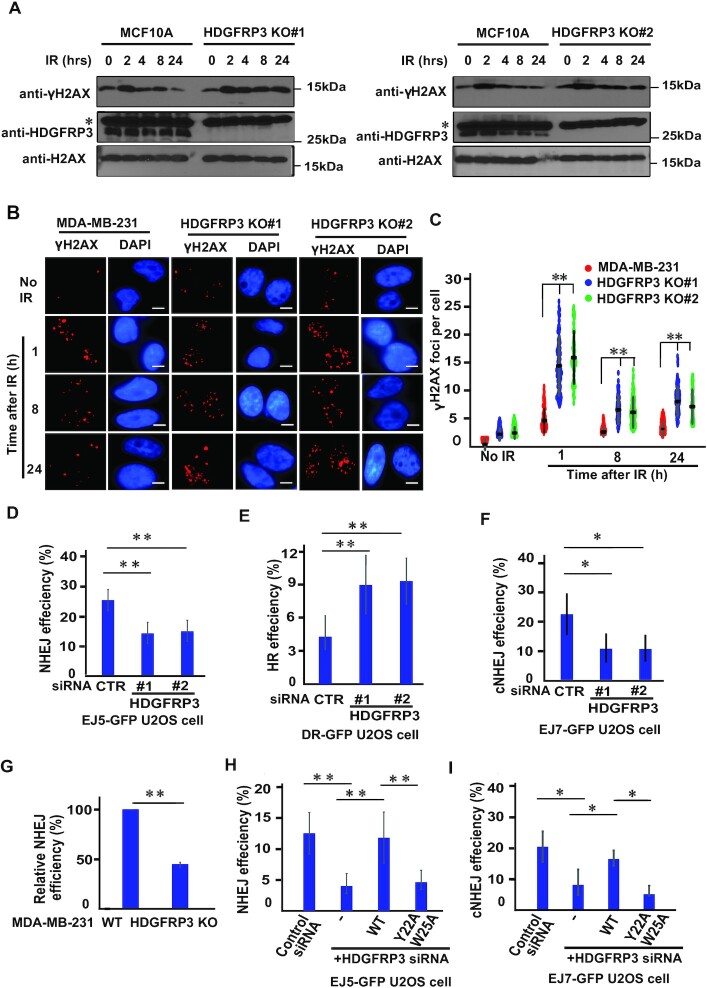
HDGFRP3 participates in DNA repair. (**A**) HDGFRP3 loss results in genomic instability. MCF10A and two different MCF10A-derivative HDGFRP3 KO cell lines were treated with IR (10 Gy), harvested at the indicated times, and immediately lysed in 2× Laemmli buffer. The lysates were immunoblotted with the indicated antibodies. The bands marked by an asterisk represent non-specific bands. Similar results were obtained from three biologically independent experiments. (**B**) Loss of HDGFRP3 impairs DNA repair. Analysis of the kinetics of IR-induced γH2AX foci in MDA-MB-231 or MDA-MB-231-derivative HDGFRP3 KO cells treated with IR (2 Gy) followed by recovery at the indicated time points. Representative images of immunofluorescent staining are shown. Scale bar, 10 μm. (**C**) Quantification of γH2AX foci in (B). γH2AX foci in these cells were quantified (at least 100 cells were counted for each of three independent experiments). Data are represented as the mean ± S.E (*n* = 3). **P < 0.01. (**D, E**) HDGFRP3 participates in DSB repair. At 24 h after transfection of HDGFRP3 siRNA, EJ5-GFP U2OS or DR-GFP U2OS cells were transfected with an *I-SceI* plasmid and pcDNA3.1-mCherry at a 9:1 ratio for another 48 h. The GFP+ and mCherry+ cell population was quantitated and the NHEJ or HR rate represented as GFP+/mCherry+. Data are represented as the mean ± S.E. (*n* = 3). **P < 0.01. (**F**) HDGFRP3 inactivation impairs cNHEJ repair. After HDGFRP3 depletion using siRNA for 24 h, EJ7-GFP-U2OS cells were transfected with mCherry and two sgRNAs, 7a and 7b at a 1:9 ratio for another 48 h. The GFP+ and mCherry+ cell population was quantitated and the cNHEJ rate represented as GFP+/mCherry+. Data are represented as the mean ± S.E. (*n* = 3). *P < 0.05. (**G**) Random plasmid integration assay was performed (*n* = 3). MDA-MB-231 and its derived HDGFRP3 KO cells were transfected with linearized pEGFP-C1 (Clontech). Cells were then grown in selection media containing G418 for 10 days, and colony numbers were determined. Data are represented as the mean ± S.E. (*n* = 3). **P < 0.01. (**H**) A defect in interaction between 53BP1 and HDGFRP3 impairs NHEJ repair. EJ5-GFP-U2OS cells were reconstituted with siRNA-resistant WT or Y22AW25A mutant of SFB-tagged HDGFRP3. After HDGFRP3 depletion using siRNA for 24 h, these cells were transfected with *I-SceI* plasmid and pcDNA3.1-mCherry at a 9:1 ratio for another 48 h. The GFP+ and mCherry+ cell population was quantitated and the NHEJ rate represented as GFP+/mCherry+. Data are represented as the mean ± S.E. (*n* = 3). **, *P* < 0.01. (**I**) A defect in interaction between 53BP1 and HDGFRP3 impairs cNHEJ repair. EJ7-GFP-U2OS cells were reconstituted with siRNA-resistant WT or Y22AW25A of SFB-tagged HDGFRP3. After HDGFRP3 depletion using siRNA for 24 h, these cells were transfected with mCherry and two sgRNAs, 7a and 7b at 1:9 ratio for another 48 h. The GFP+ and mCherry+ cell population was quantitated and the cNHEJ rate represented as GFP+/mCherry+. Data are represented as the mean ± S.E. (*n* = 3). *P < 0.05.

The HDGFR family members, e.g. HDGFRP2 and PSIP1, are involved in DNA damage and repair (55,56), highlighting the possible participation of HDGFRP3 in this process. To investigate this, cells were subjected to IR treatment, and western blotting was performed to detect the γH2AX signal. The γH2AX signal determined by immunoblotting declined markedly between 8 h and 24 h following IR in WT MCF10A (Figure 4A, Supplemental Figure S11C), whereas the kinetics of γH2AX loss was slower in two different MCF10A derivative HDGFRP3 KO cell lines at 8 and 24 h post IR (Figure 4A, Supplemental Figure S11C). Similar results were obtained from MDA-MB-231-derivative HDGFRP3 KO cells (Supplemental Figures S3A, S12B). These data indicate that the DNA repair capacity is severely impaired in HDGFRP3 KO cells after DNA damage. Consistent with the γH2AX immunoblotting signal, the number of immunofluorescent γH2AX foci declined in WT MDA-MB-231 cells at 8 h and 24 h after irradiation. The HDGFRP3 loss did not affect the initial formation of γH2AX foci, but drastically attenuated the resolution of γH2AX foci at all time-points compared to control cells following IR (Figure 4B, C). In addition, at 8 and 24 h after irradiation, 40% and 31% of γH2AX foci were resolved in HDGFRP3 KO cells, while 49% and 62% of γH2AX foci resolved in MCF10A cells, respectively. Similar results were obtained in ES2 HDGFRP3 KO ovarian cancer cells (Supplemental Figure S3B). Thus, the changes in the kinetics of the γH2AX signal indicates that HDGFRP3 is involved in DNA damage and repair. To further assess whether the observed defect in persistence of γH2AX is associated with the defect of the 53BP1–HDGFRP3 interaction, MDA-MB-231-derivative HDGFRP3 KO cells were reconstituted with sgRNA-resistant WT or Y22AW25A of SFB-tagged HDGFRP3. No difference in fluorescence intensity of FLAG signals, or infection efficiency of these plasmids was observed (Supplemental Figure S3C). As shown in Supplemental Figure S3D-S3E, the expression of WT HDGFRP3 reduced the persistence of γH2AX foci in HDGFRP3 KO cells, whereas the expression of HDGFRP3 Y22AW25A showed slow resolution of γH2AX foci in HDGFRP3 KO cells. Moreover, at 8 and 24 h after irradiation, 52% and 69% of γH2AX foci were resolved in HDGFRP3 KO cells reconstituted with WT HDGFRP3, while 34% and 46% of γH2AX foci resolved in HDGFRP3 KO cells reconstituted with HDGFRP3 Y22AW25A, respectively. These results indicate that a defect in the HDGFRP3–53BP1 interaction leads to persistence of γH2AX foci after DNA damage.

To explore the functional significance of HDGFRP3 in DSB repair, we analyzed the activity of the two DSB repair pathways, HR and NHEJ, using the previously described DR-GFP and EJ5-GFP reporter systems, respectively (44,45). As shown in Figure 4D and E, knockdown of HDGFRP3 in the EJ5-GFP reporter containing cells caused a significant reduction of the GFP-positive compared with the control cells, whereas knockdown of HDGFRP3 exhibited increased HR repair activity when assessed using the DR-GFP system (Figure 4D, E). Importantly, knockdown of HDGFRP3 does not affect cell cycle progression, transfection efficiency or *I-SceI* expression (Supplemental Figure S4A–D). Because classical NHEJ (cNHEJ) represents the predominant DSB repair pathway in mammalian cells, involves no or limited DNA end processing (57), we speculated that HDGFRP3 may be required for cNHEJ repair. Thus, we performed EJ7-GFP reporter assay (46) and found that HDGFRP3 inactivation caused a significant decrease in distal end joining, indicating that HDGFRP3 plays a role in cNHEJ repair (Figure 4F, Supplemental Figure S4E, F). To further confirm that HDGFRP3 participates in NHEJ repair, a random plasmid integration assay was used. Consistent with the NHEJ reporter assay (Figure 4D), HDGFRP3 depleted cells displayed reduced NHEJ repair as assessed by the plasmid-based integration assay (Figure 4G). To assess whether the HDGFRP3–53BP1 interaction is critical for HDGFRP3 in NHEJ and cNHEJ repair. EJ5-GFP-U2OS cells or EJ7-GFP-U2OS were reconstituted with siRNA-resistant WT or Y22AW25A mutant of SFB-tagged HDGFRP3, to express exogenous HDGFRP3 when the endogenous HDGFRP3 is depleted by siRNA. Reconstitution of WT HDGFRP3 restores NHEJ and cNHEJ repair activity, while the Y22AW25A mutant of HDGFRP3 failed to do so, indicating that a defect in the HDGFRP3–53BP1 interaction impairs NHEJ and cNHEJ repair (Figure 4H-I, Supplemental Figure S4G–J). These results indicate that HDGFRP3 is required for efficient repair of DSBs specifically through the cNHEJ pathway.

### HDGFRP3 regulates 53BP1 recruitment at sites of DNA breaks

**Figure 5.**
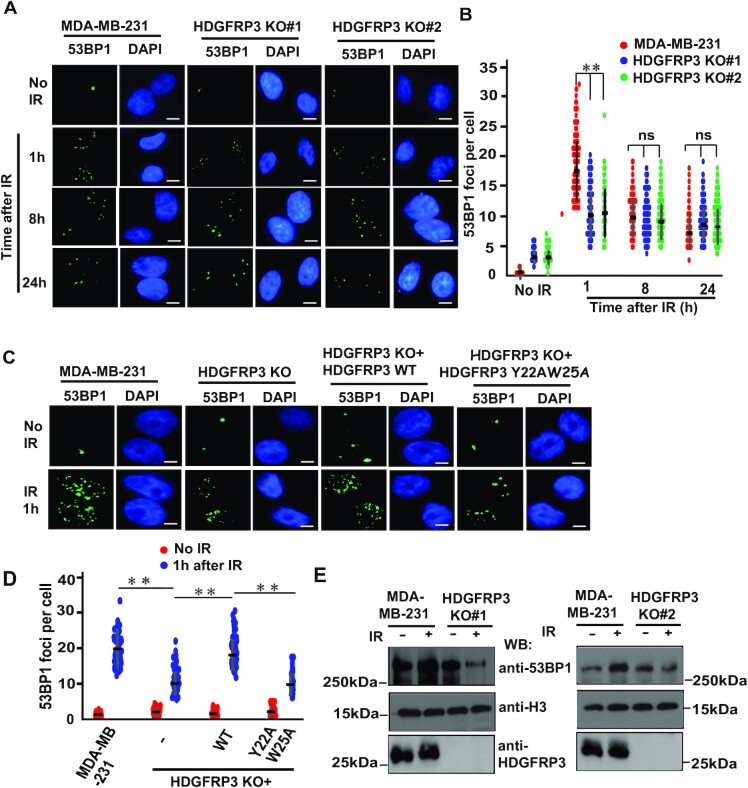
HDGFRP3 regulates 53BP1 recruitment at sites of DNA breaks. (**A**) Loss of HDGFRP3 impairs 53BP1 recruitment at DSB sites. Analysis of IR-induced 53BP1 foci kinetics in MDA-MB-231 and MDA-MB-231-derivative HDGFRP3 KO cells treated with IR (10 Gy) followed by recovery at the indicated time points. Representative images are shown by immunofluorescent staining. Scale bar, 10 μm. (**B**) Quantification of the results in (A). 53BP1 foci in these cells were quantified (at least 100 cells were counted for each of three independent experiments). Data are represented as the mean ± S.E. (*n* = 3). **P < 0.01; ns: not significant. (**C**) A defect in the interaction between 53BP1 and HDGFRP3 impairs 53BP1 recruitment at DSBs following IR. HDGFRP3 KO MDA-MB-231 cells were reconstituted with sgRNA-resistant WT or Y22AW25A mutant of SFB-tagged HDGFRP3. These cells were mock-treated or treated with IR (10 Gy). Immunofluorescence was performed using the 53BP1 antibody (11,15) at 1 h after IR. Representative images are shown by immunofluorescent staining. Scale bar, 10 μm. (**D**) Quantification of the results in (C). 53BP1 foci in these cells were quantified (at least 100 cells were counted for each of three independent experiments). Data are represented as the mean ± S.E. (*n* = 3). **, *P* < 0.01. (**E**) HDGFRP3 is required for 53BP1 chromatin loading following IR. MDA-MB-231 or MDA-MB-231-derivative HDGFRP3 KO cells were harvested at 1 h after IR treatment (10 Gy). The chromatin fractions were prepared and immunoblotted with the indicated antibodies. Three biologically independent experiments were performed, with similar results obtained.

As knockdown of HDGFRP3 decreases NHEJ activity (Figure 4D), we next tested whether HDGFRP3 regulates the presence of the NHEJ factor 53BP1 at sites of DNA break. 53BP1 foci were detected in MDA-MB-231 and its derivative HDGFRP3 KO cells following IR exposure (Figure 5A, B). Whereas irradiating MDA-MB-231 cells triggered efficient 53BP1 focus formation, the number of 53BP1 foci per cell decreased at the early timepoint (1 and 2 h) in two different HDGFRP3 KO cell lines (Figure 5A, B, Supplemental Figure S5A, B). Moreover, we did not observe a significant difference in the number of 53BP1 foci/per cell between the MDA-MB-231 and the derivative HDGFRP3 KO cells at 4, 8, and 24 h after irradiation (Figure 5A-B, Supplemental Figure S5A, B). Similar results were obtained in ES2 HDGFRP3 KO ovarian cancer cells (Supplemental Figure S5C). The observed decrease in 53BP1 foci formation upon HDGFRP3 deficiency suggests that loss of HDGFRP3 curtails 53BP1 recruitment at DSBs. To assess whether the HDGFRP3–53BP1 interaction is critical for 53BP1 recruitment at sites of DSB, MDA-MB-231-derivative HDGFRP3 KO cells were reconstituted with sgRNA-resistant WT or Y22AW25A mutant of SFB-tagged HDGFRP3. As shown in Figure 5C-D, reconstitution of WT HDGFRP3 rescues the 53BP1 recruitment at DSB site. In contrast, the Y22AW25A mutant of HDGFRP3 did not rescues the 53BP1 recruitment at DSB sites, indicating that indeed defect in the HDGFRP3–53BP1 interaction impairs 53BP1 recruitment at DSB site.

We next examined the IR-induced accumulation of 53BP1 protein on chromatin. Chromatin-bound 53BP1 signals was decreased in two different MDA-MB-231-derivative HDGFRP3 KO cell lines at 1 h following IR (Figure 5E, Supplemental Figure S11D). Additionally, there was a decrease in the recruitment of 53BP1 to chromatin in MCF10A-derivative HDGFRP3 KO and ES2-derivative HDGFRP3 KO cells compared with controls at 1 h following IR (Supplemental Figures S5D, E, S12C, D). Loss of HDGFRP3 in both MCF10A and MDA-MB-231 cell lines did not affect cell cycle progression with or without IR (Supplemental Figure S5F). Collectively, these data suggest that HDGFRP3 impacts the DNA damage response by acting upstream of 53BP1 and affecting its accumulation at sites of DSB.

### HDGFRP3 negatively regulates DNA end resection

**Figure 6.**
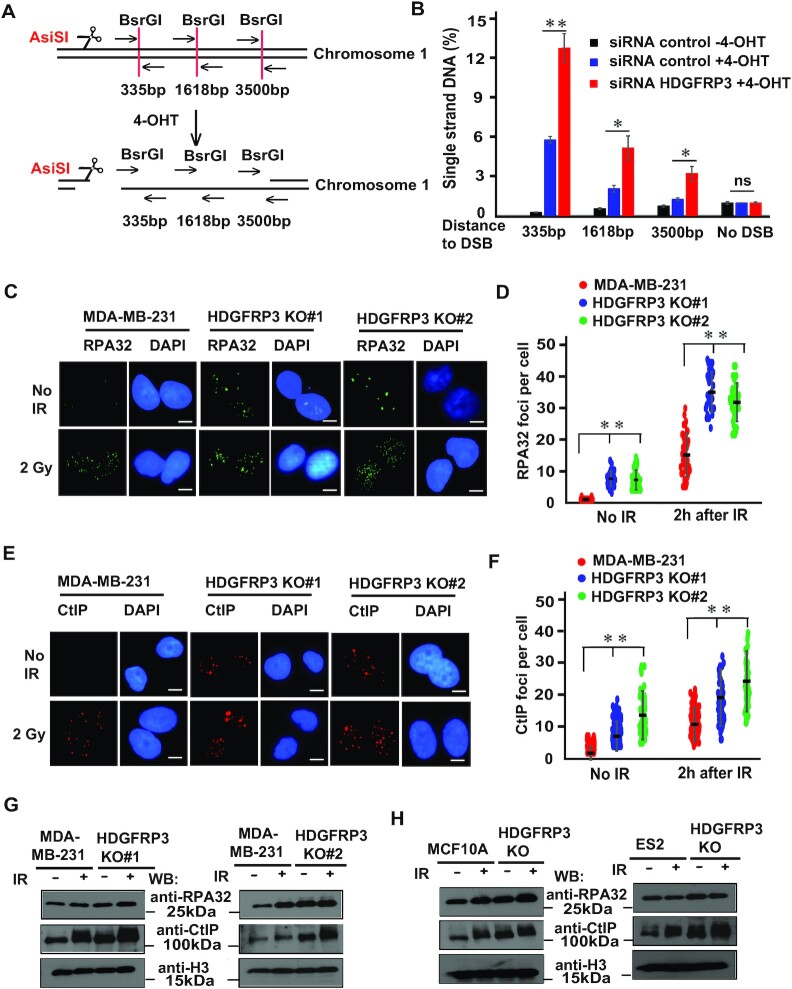
HDGFRP3 loss affects DNA end resection. (**A, B**) A schematic representation for studying the quantitative DNA resection assay based on the DIvA system (**A**). Quantitative measurement of ssDNA generation by 5’ end resection at *AsiSI*-induced DSBs (**B**). DIvA cells transfected with the indicated siRNAs were incubated with 4-OHT for 4 h or mock-treated. Genomic DNA was extracted and digested with *BsrGI*. Percentage of ssDNA intermediates at indicated sites was measured by qPCR as described in the *Methods*. Data represents mean ± S.E. *n* = 3 independent experiments. *P < 0.05, **P < 0.01; ns, not significant. (**C**) The RPA32 foci were increased in HDGFRP3 KO cells after IR. MDA-MB-231 and MDA-MB-231-derivative HDGFRP3 KO cells were treated with or without IR (2 Gy). Immunofluorescence was performed using the RPA32 antibody (MilliporeSigma) at 2 h after IR. Representative images are shown by immunofluorescent staining. Scale bar, 10 μm. (**D**) Quantification of the results in (C). RPA32 foci were quantified (at least 100 cells were counted for each of three independent experiments). Data are represented as the mean ± S.E. (*n* = 3). **P < 0.01. (**E**) The CtIP foci were increased in HDGFRP3 KO cells after IR. MDA-MB-231 and derivative HDGFRP3 KO cells were treated with or without IR (2 Gy). Immunofluorescence was performed using the CtIP antibody (Active Motif) at 2 h after IR. Representative images are shown by immunofluorescent staining. Scale bar, 10 μm. (**F**) Quantification of the results in (E). CtIP foci were quantified (at least 100 cells were counted for each of three independent experiments). Data are represented as the mean ± S.E. (*n* = 3). **, *P* < 0.01. (**G**) Loss of HDGFRP3 increases the occupancy of RPA32 and CtIP on damaged chromatin. MDA-MB-231 and two different derivative HDGFRP3 KO cell lines were harvested at 1 h after IR treatment. The chromatin fractions were prepared and immunoblotted with the indicated antibodies. Similar results were obtained from three biologically independent experiments. (**H**) Loss of HDGFRP3 increases the occupancy of RPA32 and CtIP on damaged chromatin. MCF10A/ES2 and MCF10A/ES2-derivative HDGFRP3 KO cells were harvested at 1 h after IR treatment. The chromatin fractions were prepared and immunoblotted with the indicated antibodies. Three biologically independent experiments were performed, with similar results obtained.

Given that 53BP1 restrains DNA end resection to promote NHEJ repair, we examined whether HDGFRP3 is involved in DSB resection. To this end, we performed a quantitative DNA resection assay based on the DIvA system (58). Briefly, DIvA cells pretreated with indicated siRNAs were incubated with 4-Hydroxytamoxifen (4-OHT). Genomic DNA was extracted and digested with *BsrGI* (Figure 6A). The percentage of ssDNA intermediates at indicated sites was quantified by qPCR (Figure 6B). An irrelevant site that spans a *HindIII* restriction site was included as a negative control. Notably, deficiency of HDGFRP3 reproducibly led to increased abundance of ssDNA intermediates (Figure 6B), results that are in line with the role of HDGFRP3 in limiting DSB resection. We next asked if the loss of HDGFRP3 affected DNA end resection by investigating RPA32 foci formation using immunofluorescence. Indeed, RPA32 foci formation was enhanced in two different MDA-MB-231-derivative HDGFRP3 KO cell lines and was further increased after IR in HDGFRP3 KO cells (Figure 6C, D). Similar results were obtained in ES2 HDGFRP3 KO ovarian cancer cells, in which the number of RPA32 foci per cell was increased, and markedly further increased after IR exposure (Supplemental Figure S6A). Further corroborating these findings, we found that the loss of HDGFRP3 upregulated RPA32 protein levels on the chromatin in HDGFRP3 KO cells, and further increased accumulation of RPA32 protein on the chromatin in response to IR, compared with WT MDA-MB-231, MCF10A and ES2 cells (Figure 6G, H, Supplemental Figure S11E, F).

DSB resection is initiated by CtIP, together with the MRN complex. Given the effect of HDGFRP3 on DNA end resection, we wondered if HDGFRP3 functions at the level of CtIP. Examining the recruitment of CtIP at DSB sites, we found that CtIP foci formation was increased in two different MDA-MB-231-derivative HDGFRP3 KO cell lines and was markedly increased after IR in HDGFRP3 KO cells (Figure 6E, F). Additionally, similar results were obtained in ES2 HDGFRP3 KO cells after IR (Supplemental Figure S6B). In accordance with this, we next examined the accumulation of CtIP protein on the chromatin in these cells. Loss of HDGFRP3 resulted in elevated CtIP levels in HDGFRP3 KO cells and exhibited largely increased CtIP protein on the chromatin following IR, compared with WT MDA-MB-231, MCF10A, and ES2 cells (Figure 6G, H, Supplemental Figure S11E, F). Taken together, these results demonstrate that HDGFRP3 promotes NHEJ repair by negatively regulating DNA end resection.

Establishing the role of HDGFRP3 in limiting DNA end resection, we next tested whether the HDGFRP3–53BP1 interaction is important for its anti-resection function. We re-expressed HDGFRP3 WT and the Y22AW25A mutant in HDGFRP3 KO cells and then examined RPA32 and CtIP foci. As shown in Supplemental Figure S6C-S6F, the HDGFRP3 WT, but not the HDGFRP3 Y22AW25A mutant, inhibited RPA32 and CtIP foci following DNA damage. Moreover, the expression of WT HDGFRP3 reduced the chromatin-bound RPA32 and CtIP protein level in HDGFRP3 KO cells. In contrast, the expression of HDGFRP3 Y22AW25A mutant increased the chromatin-bound RPA32 and CtIP protein level in HDGFRP3 KO cells (Supplemental Figures S6G, S12E), indicating that the HDGFRP3–53BP1 interaction is critical for inhibition of DNA end resection.

### HDGFRP3 regulates DNA repair pathway choice

**Figure 7.**
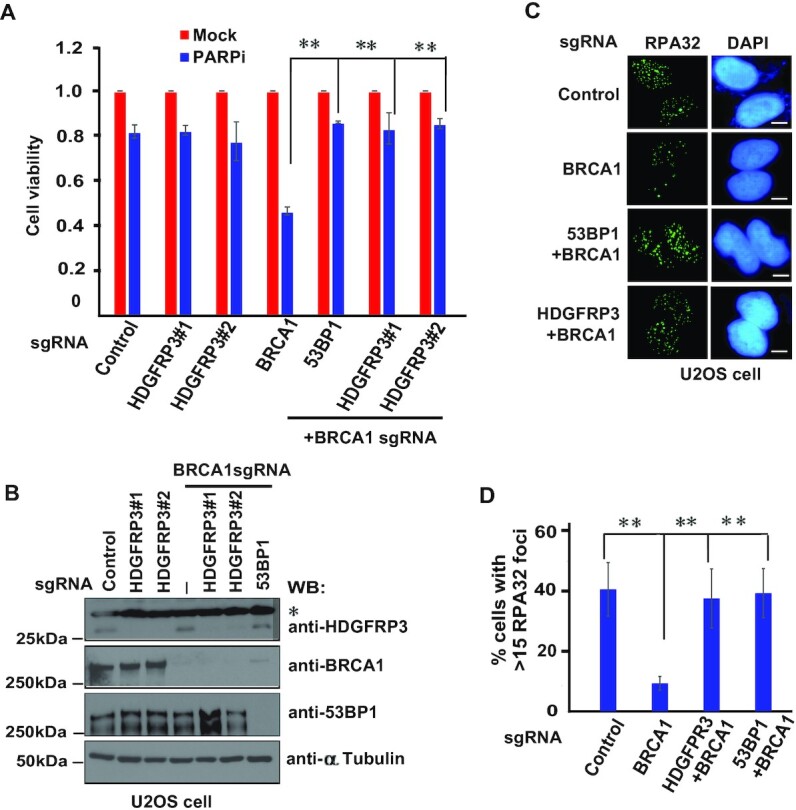
HDGFRP3 regulates DNA repair pathway choice. (**A**) Loss of HDGFRP3 renders BRCA1-deficient cells resistant to PARP inhibition. U2OS cells were infected with the indicated sgRNAs and treated with 1 μM PARPi (Olaparib). Colony formation was quantified relative to colonies formed in untreated cells from the same setting. Data are represented as the mean ± S.E. (n = 3). **P < 0.01. (**B**) Western blot analysis of HDGFRP3, BRCA1 and 53BP1 expression in (A). The bands marked by an asterisk represent non-specific bands. (**C**) HDGFRP3 prevents end resection in the absence of BRCA1. U2OS cells were transfected with the indicated sgRNAs. At 2 h post-irradiation (10 Gy), cells were processed for RPA32 immunofluorescence staining. Scale bar, 10 μm. (**D**) Quantification of the results in (C). RPA32 foci were quantified (at least 100 cells were counted for each experiment, three independent experiments). Data are represented as the mean ± S.E. (n = 3). **P < 0.01.

Because HDGFRP3 promotes NHEJ repair and represses DNA end resection, we speculated that HDGFRP3 may regulate DNA repair pathway choice. Indeed, HDGFRP3 depletion rendered BRCA1-deficient U2OS cells resistant to PARP inhibition (Figure 7A, B). Similar results were shown for HDGFRP3 deficiency that conferred PARPi resistance in BRCA1 depleted MDA-MB-231 cells (Supplemental Figure S7A, B). RPA32 binding to resected DNA ends is a key step in HR repair, therefore the formation of RPA32 foci can be used as a key indicator. As shown in Figure 7C, D, IR-induced RPA32 foci were reduced in BRCA1-depleted U2OS cells, but they were re-established in BRCA1/HDGFRP3 co-depleted U2OS cells, indicating that similar to 53BP1, co-depletion of HDGFRP3 together with BRCA1 promotes DNA end resection and HR repair. Furthermore, RPA32 foci were also restored in the absence of HDGFRP3 and BRCA1 in both U2OS and MDA-MB-231 cells after treatment with PARPi (Supplemental Figure S7C–F), indicating that HDGFRP3 antagonizes BRCA1 to regulate DSB repair pathways.

### HDGFRP3 and 53BP1 differentially interact with methylated H4K20 following DNA damage

**Figure 8.**
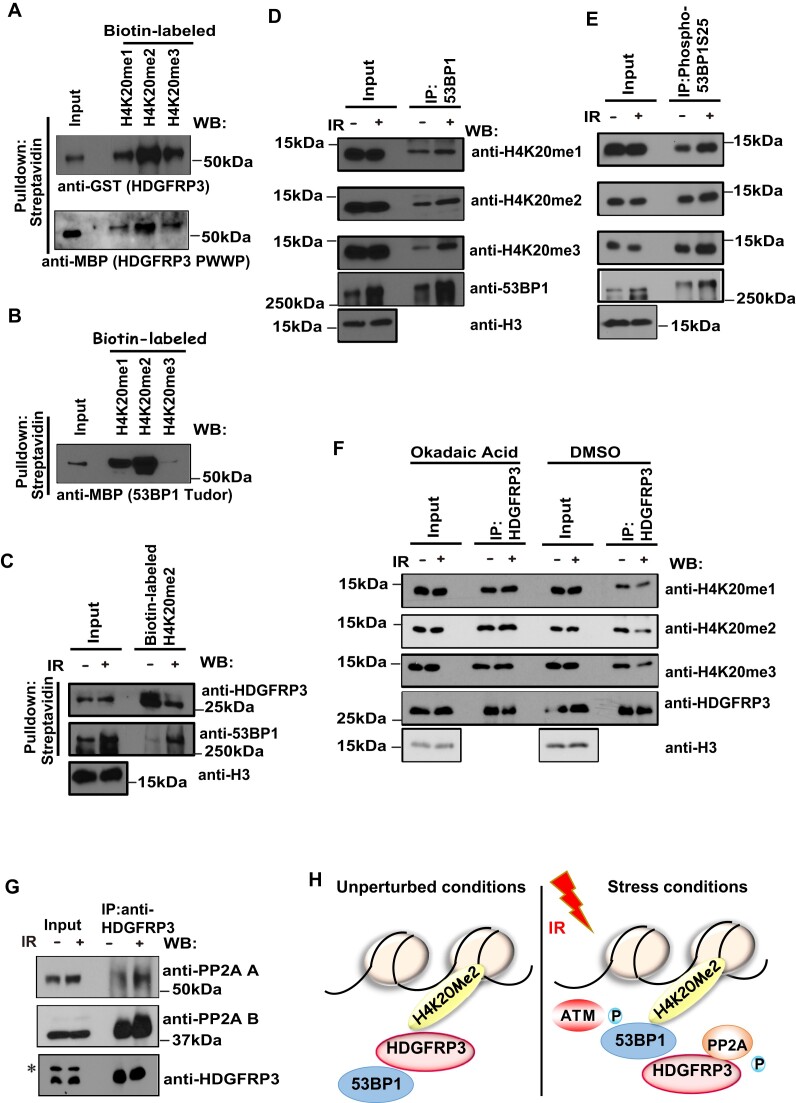
HDGFRP3 and 53BP1 interact with methylated H4K20 in a differential manner following DNA damage. (**A**) HDGFRP3 binds to methylated H4K20. Biotin-conjugated histone peptides were incubated with bacterially expressed GST-HDGFRP3 or MBP-HDGFRP3 PWWP proteins, followed by pull-down with streptavidin beads. Western blotting was performed with the indicated antibodies. Similar results were obtained from three biologically independent experiments. (**B**) The Tudor domain of 53BP1 binds to methylated H4K20. Biotin-conjugated histone peptides were incubated with bacterially expressed MBP-53BP1 Tudor domain protein, followed by pull-down with streptavidin beads. Western blotting was performed with the indicated antibodies. Three biologically independent experiments were performed, with similar results obtained. (**C**) HDGFRP3 and 53BP1 interact with Biotin-H4K20me2 in a differential manner following DNA damage. MCF10A cells were harvested at 1 h following treatment with IR (10 Gy). Chromatin fractions were extracted and added to Biotin-H4K20me2 peptides that were immobilized on streptavidin beads. Beads were washed and boiled, and then subjected to Western blotting using the indicated antibodies. Similar results were obtained from three biologically independent experiments. (**D**) The binding affinity of methylated H4K20 with 53BP1 was increased following DNA damage. MCF10A cells were harvested at 1 h following treatment with IR (10 Gy). Chromatin fractions were extracted and immunoprecipitated with a 53BP1 antibody (Santa Cruz). Immunoprecipitates were blotted using the indicated antibodies. Three biologically independent experiments were performed, with similar results obtained. (**E**) The binding affinity of methylated H4K20 with phosphorylated 53BP1 was increased following DNA damage. MCF10A cells were harvested at 1 h following treatment with IR (10 Gy). Chromatin fractions were extracted and immunoprecipitated with a Phospho-53BP1 (Ser25) antibody (Bethyl Laboratories). Immunoprecipitates were blotted using the indicated antibodies. Similar results were obtained from three biologically independent experiments. (**F**) HDGFRP3 dissociates from methylated H4K20 after IR, which is regulated by okadaic acid. MCF10A were either left untreated, irradiated with 10 Gy or treated with okadaic acid (0.5 μM) at 1 h after IR (10 Gy). One hour later, chromatin fractions were extracted and immunoprecipitated with a HDGFRP3 antibody (Proteintech). Immunoprecipitates were blotted using the indicated antibodies. Three biologically independent experiments were performed, with similar results obtained. (**G**) HDGFRP3 binds PP2A in a manner dependent on DNA damage. Immunoprecipitation (IP) of HDGFRP3 (Proteintech) from untreated or irradiated (10 Gy, 1 h recovery) HEK293T cells followed by immunoblotting with the HDGFRP3 (Assay Biotech) and the PP2A-A or PP2A-B antibodies (Cell Signaling Technology). Similar results were obtained from three biologically independent experiments. (**H**) A working model representing the dynamic 53BP1-methylated H4K20–HDGFRP3 complex that regulates the recruitment of 53BP1 to DNA damage sites. Please see details in the Discussion.

The PWWP domains have been suggested to function as methyl-lysine histone-binding modules (55). To analyze the binding of HDGFRP3 PWWP domain to specific histone marks, biotin-conjugated methylated histone H4 peptides were incubated with bacterially expressed GST-HDGFRP3 or MBP-HDGFRP3 PWWP, immobilized on streptavidin beads, and analyzed by immunoblotting. As shown in Figure 8A, Supplemental Figure S11G, HDGFRP3 or HDGFRP3 PWWP had a high affinity for biotinylated histone H4K20me2, and less affinity for H4K20me1 and H4K20me3 peptides *in vitro*. 53BP1 has been shown to recognize mono-methylated and di-methylated histones via its Tudor domain, which is vital for its recruitment to DSB sites (27,28,59). Indeed, the Tudor domain of 53BP1 preferentially interacted with H4K20me2, and to a lesser extent with H4K20me1 and H4K20me3 (Figure 8B, Supplemental Figure S11H). We next examined the functional significance of HDGFRP3 and 53BP1 binding to H4K20me2 before and after DNA damage. We performed a biotin-H4K20me2 peptide pulldown assay and showed that the interaction between HDGFRP3 and H4K20me2 peptide decreased after DNA damage. In contrast, the binding affinity of H4K20me2 with 53BP1 was increased after IR (Figure 8C, Supplemental Figure S11I), suggesting that HDGFRP3 and 53BP1 differentially interact with H4K20me2 following DNA damage. To further determine the regulation of methylated H4K20 with HDGFRP3 and 53BP1 *in vivo*, immunoprecipitation (IP) reactions were conducted in chromatin fraction with anti-53BP1 or anti-HDGFRP3 antibodies, followed by immunoblotting with antibodies to methylated H4K20. We observed that the interaction between 53BP1 and methylated H4K20 increased after IR treatment (Figure 8D, Supplemental Figure S11J). 53BP1 is phosphorylated on its 28 S/TQ sites at its N terminus by ATM. An IP reaction was performed in chromatin fraction with a phospho-53BP1 (Ser25) antibody, followed by immunoblotting with antibodies to methylated H4K20. We observed that the interaction between phospho-53BP1 and methylated H4K20 further increased after IR treatment in MCF10A and its derivative HDGFRP3 KO cells (Figure 8E, Supplemental Figures S11K, S8A, S12F), indicating that HDGFRP3 loss has no effect on the increased interaction between phospho-53BP1 and methylated H4K20 after IR treatment. In addition, IP reactions were performed in chromatin fractions with an anti-HDGFRP3 antibody, followed by immunoblotting with antibodies to methylated H4K20. Interestingly, we observed that HDGFRP3 dissociated from methylated H4K20 after IR treatment (Figure 8F, Supplemental Figure S11L). To further confirm the specificity of the 53BP1–H4K20me2 and HDGFRP3–H4K20me2 interaction following DNA damage, the PLA assay was performed in U2OS cells. In agreement with the results from endogenous IP (Figure 8D, F), a significantly higher number of PLA dots per nucleus was observed with the H4K20me2-53BP1 interaction following DNA damage (Supplemental Figure S8B, C). In addition, the cells used for detection of the H4K20me2–HDGFRP3 interaction displayed much fewer dots per nucleus after treatment with IR (Supplemental Figure S8D, E).

We next examined whether phosphorylation of HDGFRP3 is mediated by IR and/or ATM. Using the bacterially expressed MBP-53BP1 tudor domain protein, we showed that its interaction with SFB-HDGFRP3 was increased after IR. However, it was not notably changed with combined IR and ATMi treatment (Supplemental Figures S8F, S12G), indicating that phosphorylation of HDGFRP3 and its association with 53BP1 may not be regulated by the ATM kinase. In addition, the biotin-H4K20me2 peptide pulldown assay showed that the dissociation of H4K20me2 with HDGFRP3 was not altered after ATMi treatment (Supplemental Figures S8G, S12H), suggesting that ATM kinase might not control the dissociation of HDGFRP3 with H4K20me2 following DNA damage. To characterize the mechanism of dissociation of HDGFRP3 from H4K20me, whether it is regulated by phosphorylation and dephosphorylation by related phosphatases, we performed a TAP-MS assay to identify the phosphatase that dephosphorylates HDGFRP3 following DNA damage. The A subunit of PP2A complex was identified as a potential partner of HDGFRP3 (Supplementary Figure S1G). Therefore, we examined the interaction between HDGFRP3 and the subunits of PP2A, including PP2A-A, PP2A-B, with or without IR, by endogenous IP. Indeed, the interaction between PP2A-A or B and HDGFRP3 was enhanced in these cells after IR (Figure 8G, Supplemental Figure S11M), indicating that, in response to DNA damage, PP2A-A or B may dephosphorylate HDGFRP3. In agreement with the results from endogenous IP (Figure 8G, Supplemental Figure S11M), a significantly higher number of PLA dots per nucleus was observed with the PP2A-A or B and HDGFRP3 interaction following DNA damage (Supplemental Figure S8H–K). Furthermore, we treated cells with a protein phosphatase PP2A inhibitor okadaic acid. As shown in Figure 8F, the HDGFRP3-methylated H4K20 interaction is increased following DNA damage upon treatment with okadaic acid, indicating that HDGFRP3 dephosphorylation leads to the dissociation of HDGFRP3 with methylated H4K20. Together, these data suggest a dynamic interplay of the HDGFRP3-–H4K20me2–53BP1 complex for the recruitment of 53BP1 at DSB sites, which is likely regulated by protein phosphorylation and dephosphorylation.

## DISCUSSION

DNA DSBs are potentially lethal lesions, which are primarily repaired by NHEJ or HR. 53BP1 plays a critical role in the preservation of genomic integrity (60) and the recruitment of 53BP1 at sites of DNA damage represses DNA end resection, thereby favoring NHEJ (61). 53BP1 is recruited to DSBs by recognizing H4K20me2 and H2AK15ub (62,63). 53BP1 has recently attracted particular attention because of its role in the pathway choice between NHEJ and HR and its relevance to PARPi treatment of BRCA1-mutant cancers.

In this study, we identified HDGFRP3 as a 53BP1-binding protein. The interaction of HDGFRP3 with 53BP1 is controlled by an ATM-dependent DNA damage response that requires 53BP1 phosphorylation. This regulation by ATM strongly reinforces the physiological importance of the 53BP1–HDGFRP3 interaction. Our data further revealed that the HDGFRP3–53BP1 interaction is mediated by the PWWP domain of HDGFRP3 and the Tudor domain of 53BP1. As a member of the family of HDGF-related proteins, HDGFRP3 was shown to be frequently upregulated in human hepatocellular carcinoma cells (43) and to govern the development of neurons in the brain (64,65). However, little is known about the function of HDGFRP3 in the DNA damage response and DNA repair. Here, we report multiple lines of evidence that HDGFRP3 is a novel component of the DSB repair machinery. First, we provide evidence that the HDGFRP3–53BP1 interaction is enhanced and localizes at sites of DSB following IR. Second, we show that HDGFRP3 participates in cNHEJ repair. Third, we demonstrate that HDGFRP3 regulates 53BP1 recruitment at DSB sites and inhibits DNA end resection. Lastly, we show that HDGFRP3 promotes NHEJ repair and antagonizes BRCA1-dependend HR repair to regulate DNA repair pathway choice. Consequently, depletion of HDGFRP3 leads to both the accumulation of endogenous DSB and DNA damage hypersensitivity. Furthermore, the HDGFRP3–53BP1 interaction is required for cNHEJ repair activity, 53BP1 recruitment at DSB sites, resolution of the γH2AX signal, and inhibition of DNA end resection. Taken together, our studies reveal a new role of HDGFRP3 in facilitating DNA DSB repair.

Clearly, the regulatory mechanism of 53BP1 binding to damaged chromatin is a complex process. 53BP1 accumulation at DSBs is affected by early responsive DNA repair factors, such as the upstream regulator MDC1 (66,67). We observed that siRNA-mediated MDC1 knockdown dramatically decreased the 53BP1–HDGFRP3 interaction after IR by the PLA assay (Supplemental Figure S9A-S9C), indicating that MDC1 regulates 53BP1 recruitment and its interaction with HDGFRP3 at DNA damage sites. Furthermore, the recruitment of 53BP1 at DSBs requires its binding to H4K20me2 via its Tudor domain (27,28). However, H4K20me is buried in stacked nucleosomes, and therefore it is usually inaccessible to 53BP1 (30). JMJD2A and L3MBTL1 that also contain Tudor domains that bind to H4K20me2, are either degraded or removed by the E3 ligase RNF8 and RNF168 upon DNA damage, and thus allow exposure of H4K20 methylation for 53BP1 binding at DNA damage sites (31–33). Indeed, RNF8 is critical to expose H4K20me2 for the recruitment of 53BP1 to DNA damage sites (Supplemental Figure S9D-S9F). The PWWP domain is a methyl-lysine recognition motif that plays an important role in epigenetic regulation (68,69). Structural analysis revealed that the PWWP domain of *S. pombe*, Pdp1, binds to methylated H4K20me, which is required for Set9 chromatin localization (68,69). Here, we show that the PWWP domain of HDGFRP3 directly binds to methylated H4K20 peptides, and we further observe that HDGFRP3 dissociates from H4K20me following DNA damage. Moreover, the binding affinity of H4K20me with 53BP1 and phospho-53BP1 was increased following DNA damage, suggesting that HDGFRP3 and 53BP1 interact with H4K20me in a differential manner following DNA damage. Our current working model is that H4K20me2 is bound by HDGFRP3 under normal conditions. The DNA damage triggers the dissociation of HDGFRP3–H4K20me2 to allow the exposure of H4K20me2 for binding of 53BP1, which is likely regulated by protein phosphorylation and dephosphorylation (Figure 8H).

To explore the underlying mechanism for dissociation of HDGFRP3 from H4K20me following DNA damage, we investigated whether phosphorylation events affect the binding affinity of HDGFRP3 to methylated H4K20. The reversible phosphorylation of proteins, catalyzed by protein kinases and phosphatases, is a major mechanism for regulating DSB repair (70). The phosphorylation of HDGFRP3 is not regulated by ATM kinase because the increased interaction of HDGFRP3 and 53BP1 and the dissociation of HDGFRP3 with H4K20me2 are not altered upon IR and ATMi treatment. PP2A is known to dephosphorylate many substrates involved in the cell cycle (71). We show that the interaction between PP2A-A or B and HDGFRP3 was enhanced in cells after IR. We further show that the HDGFRP3-methylated H4K20 interaction is increased following IR upon treatment with okadaic acid (a PP2A inhibitor), suggesting that dephosphorylation of HDGFRP3 leads to the dissociation of HDGFRP3 with methylated H4K20 following IR. Thus, the impact of PP2A as potentially being responsible for HDGFRP3 dephosphorylation needs further study, by examining its role in 53BP1 interaction and on DNA damage persistence or genome (in)stability in the future.

In summary, our results reveal a dynamic interplay of the 53BP1-methylated H4K20–HDGFRP3 complex for the recruitment of 53BP1 at DSB sites. Data extracted from cBioportal (http://www.cBioportal.org) show that 29 of 35 cancer types exhibit HDGFRP3 gene amplification (Supplemental Figure S10A). Moreover, we observed a significant positive correlation between HDGFRP3 and 53BP1 transcript levels in different cancer types (Supplementary Figure S10B). Future studies should be devoted to taking advantage of this knowledge and developing strategies to design more efficient anti-cancer therapeutics to overcome therapeutic resistance.

## Supplementary Material

gkad073_Supplemental_Files

## Data Availability

The data underlying this article will be shared on reasonable request to the corresponding author.
