# Correction to ‘HDGFRP3 interaction with 53BP1 promotes DNA double-strand break repair’

**DOI:** 10.1093/nar/gkae146

**Published:** 2024-02-26

*Nucleic Acids Research*, Volume 51, Issue 5, 21 March 2023, Pages 2238–2256, https://doi.org/10.1093/nar/gkad073

The authors would like to apologize for an error in Figure 8E of their article. During figure assembly and manuscript preparation, the Western blot image for H4K20me3 was accidentally used for both the “H4K20me1” and “H4K20me3”. The original blots and corrected figure appear below. This error does not affect the results and conclusion of the article.

**The original blots**


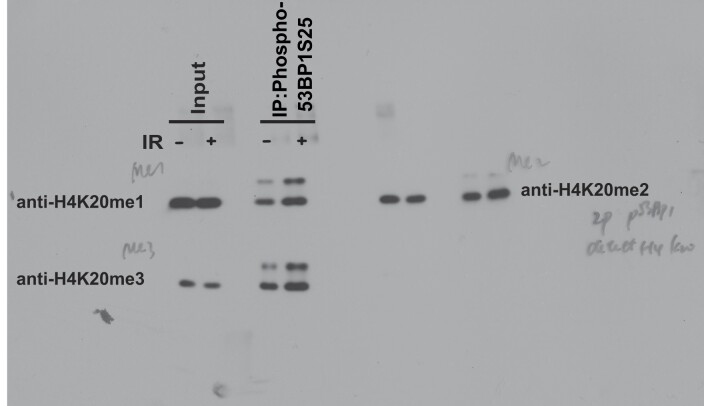


**The corrected figure**

**Figure 8.**
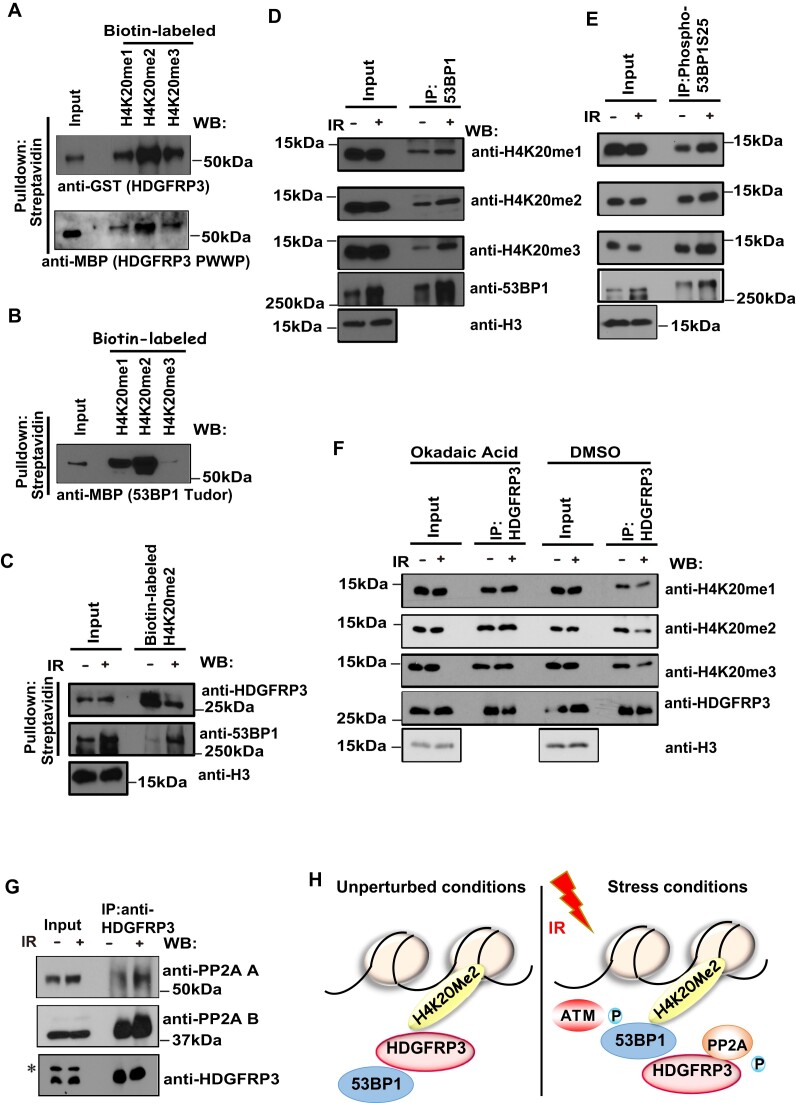
HDGFRP3 and 53BP1 interact with methylated H4K20 in a differential manner following DNA damage. (**A**) HDGFRP3 binds to methylated H4K20. Biotin-conjugated histone peptides were incubated with bacterially expressed GST-HDGFRP3 or MBP-HDGFRP3 PWWP proteins, followed by pull-down with streptavidin beads. Western blotting was performed with the indicated antibodies. Similar results were obtained from three biologically independent experiments. (**B**) The Tudor domain of 53BP1 binds to methylated H4K20. Biotin-conjugated histone peptides were incubated with bacterially expressed MBP-53BP1 Tudor domain protein, followed by pull-down with streptavidin beads. Western blotting was performed with the indicated antibodies. Three biologically independent experiments were performed, with similar results obtained. (**C**) HDGFRP3 and 53BP1 interact with Biotin-H4K20me2 in a differential manner following DNA damage. MCF10A cells were harvested at 1 h following treatment with IR (10 Gy). Chromatin fractions were extracted and added to Biotin-H4K20me2 peptides that were immobilized on streptavidin beads. Beads were washed and boiled, and then subjected to Western blotting using the indicated antibodies. Similar results were obtained from three biologically independent experiments. (**D**) The binding affinity of methylated H4K20 with 53BP1 was increased following DNA damage. MCF10A cells were harvested at 1 h following treatment with IR (10 Gy). Chromatin fractions were extracted and immunoprecipitated with a 53BP1 antibody (Santa Cruz). Immunoprecipitates were blotted using the indicated antibodies. Three biologically independent experiments were performed, with similar results obtained. (**E**) The binding affinity of methylated H4K20 with phosphorylated 53BP1 was increased following DNA damage. MCF10A cells were harvested at 1 h following treatment with IR (10 Gy). Chromatin fractions were extracted and immunoprecipitated with a Phospho-53BP1 (Ser25) antibody (Bethyl Laboratories). Immunoprecipitates were blotted using the indicated antibodies. Similar results were obtained from three biologically independent experiments. (**F**) HDGFRP3 dissociates from methylated H4K20 after IR, which is regulated by okadaic acid. MCF10A were either left untreated, irradiated with 10 Gy or treated with okadaic acid (0.5 μM) at 1 h after IR (10 Gy). One hour later, chromatin fractions were extracted and immunoprecipitated with a HDGFRP3 antibody (Proteintech). Immunoprecipitates were blotted using the indicated antibodies. Three biologically independent experiments were performed, with similar results obtained. (**G**) HDGFRP3 binds PP2A in a manner dependent on DNA damage. Immunoprecipitation (IP) of HDGFRP3 (Proteintech) from untreated or irradiated (10 Gy, 1 h recovery) HEK293T cells followed by immunoblotting with the HDGFRP3 (Assay Biotech) and the PP2A-A or PP2A-B antibodies (Cell Signaling Technology). Similar results were obtained from three biologically independent experiments. (**H**) A working model representing the dynamic 53BP1-methylated H4K20–HDGFRP3 complex that regulates the recruitment of 53BP1 to DNA damage sites. Please see details in the Discussion.

