# Supplementary material for: HDGFRP3 interaction with 53BP1 promotes DNA double-strand break repair
Source: Nucleic Acids Res. 2023 Feb 16;51(5):2238–56. doi: 10.1093/nar/gkad073 (PMC10018360; doi:10.1093/nar/gkad073)

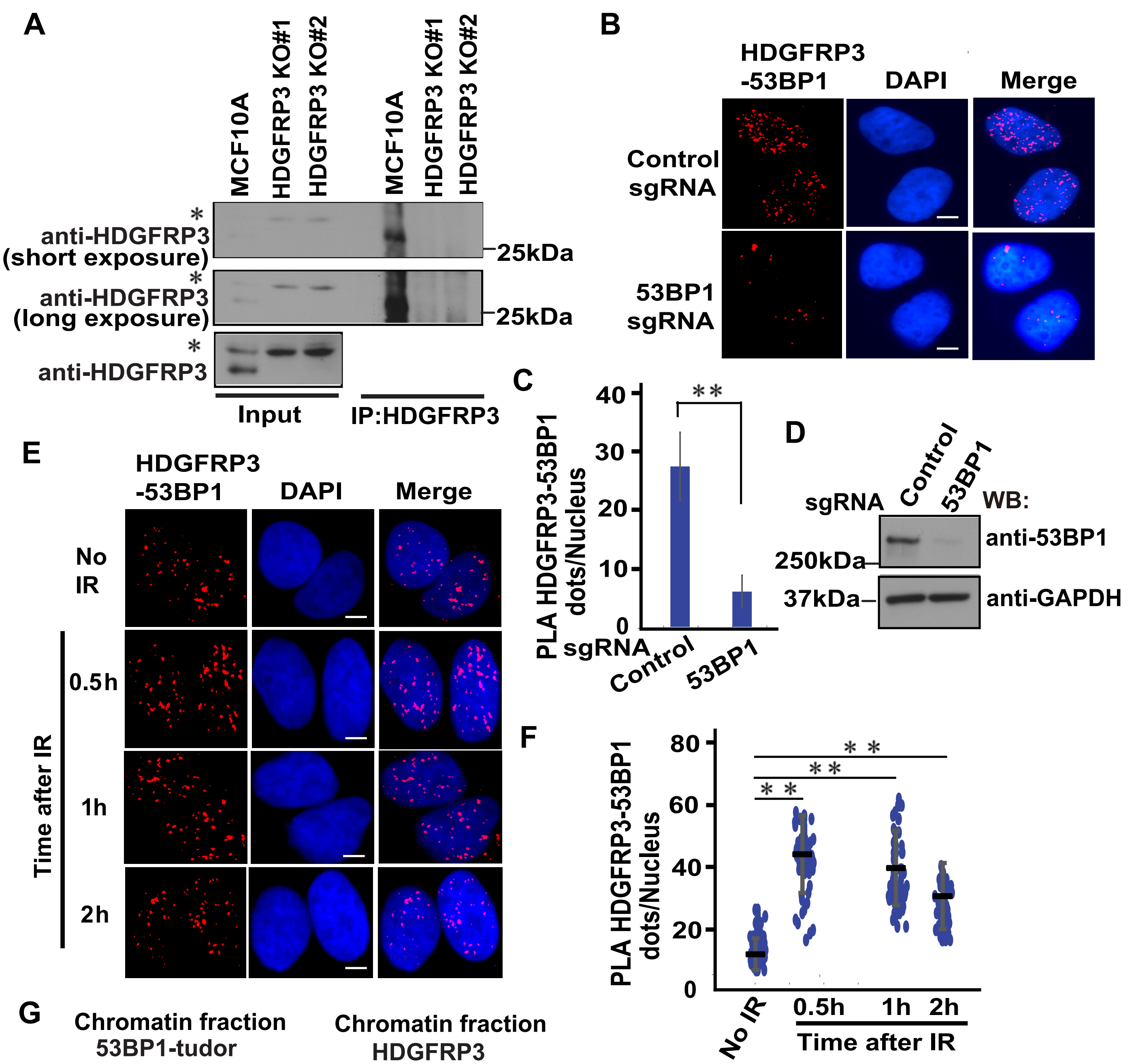

G

Chromatin fraction 53BP1-tudor	
Protein	Number of Peptides
53BP1	69
PARP1	52
RPA1	19
HDGFRP3	15
MECP2	13
MDC1	5
RIF1	1
......	

Chromatin fraction HDGFRP3	
Protein	Number of Peptides
PARP1	55
CHAF1A	23
MECP2	22
53BP1	22
HDAC1	15
HDGFRP3	13
RBBP4	13
PPP2R1A	5
......	

H

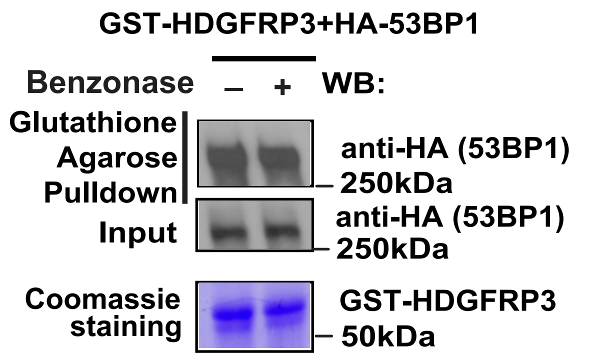

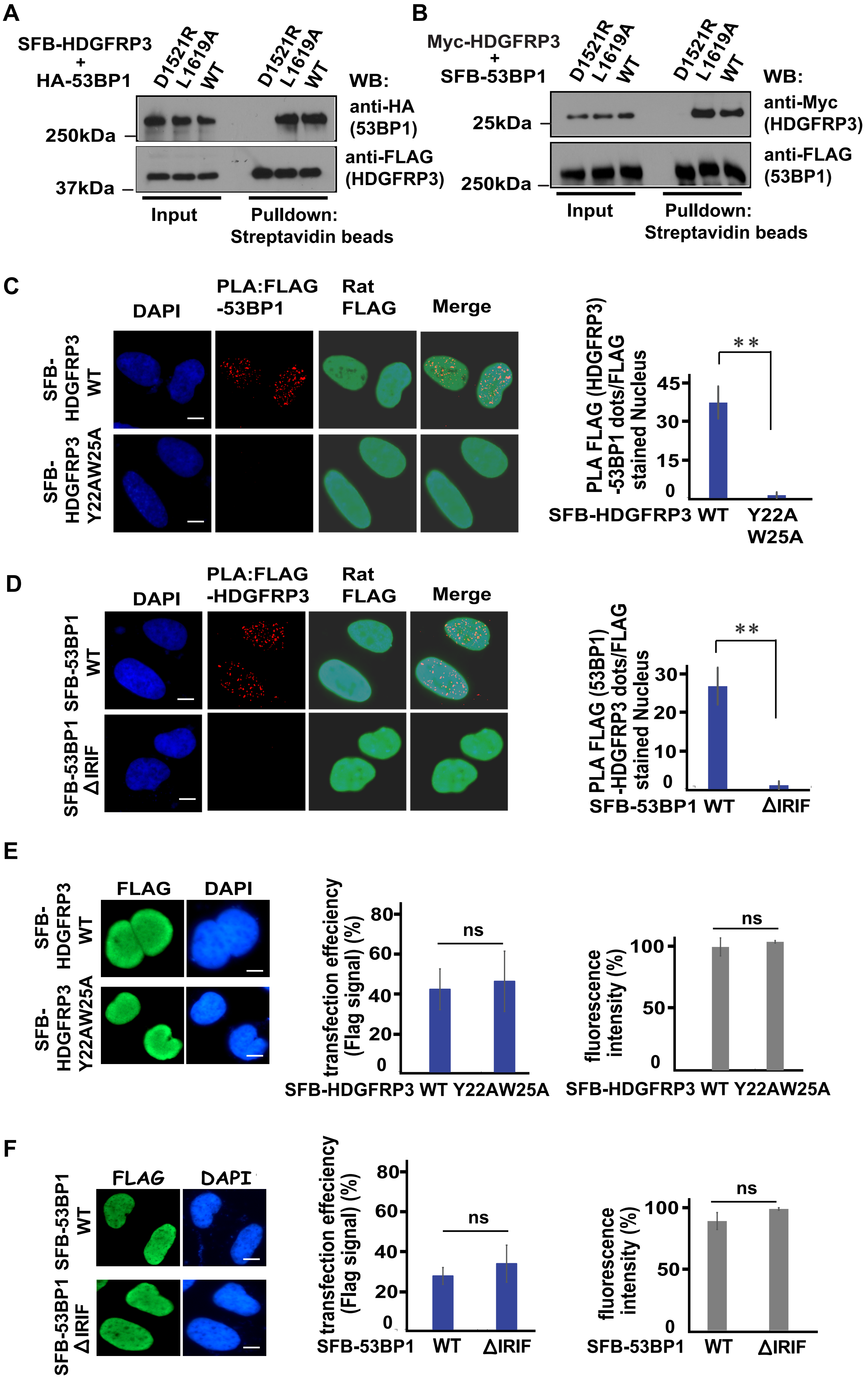
A
SFB-HDGFRP3 + HA-53BP1
D1521R
L1619A
WT
WB:
anti-HA (53BP1)
250kDa
anti-FLAG (HDGFRP3)
37kDa
Input
Pulldown: Streptavidin beads
B
Myc-HDGFRP3 + SFB-53BP1
anti-Myc (HDGFRP3)
25kDa
anti-FLAG (53BP1)
250kDa
C
DAPI
PLA:FLAG -53BP1
Rat FLAG
Merge
SFB-HDGFRP3 WT
SFB-HDGFRP3 Y22AW25A
PLA FLAG (HDGFRP3) -53BP1 dots/FLAG stained Nucleus
**
45
30
15
0
SFB-HDGFRP3 WT
Y22A W25A
D
PLA:FLAG -HDGFRP3
SFB-53BP1 WT
SFB-53BP1 ΔIRIF
PLA FLAG (53BP1) -HDGFRP3 dots/FLAG stained Nucleus
30
20
10
0
SFB-53BP1 WT
ΔIRIF
E
FLAG
DAPI
transfection effeciency (Flag signal) (%)
80
60
40
20
0
ns
SFB-HDGFRP3 WT Y22AW25A
fluorescence intensity (%)
100
50
0
F

Zhang_Supplemental Figure S3

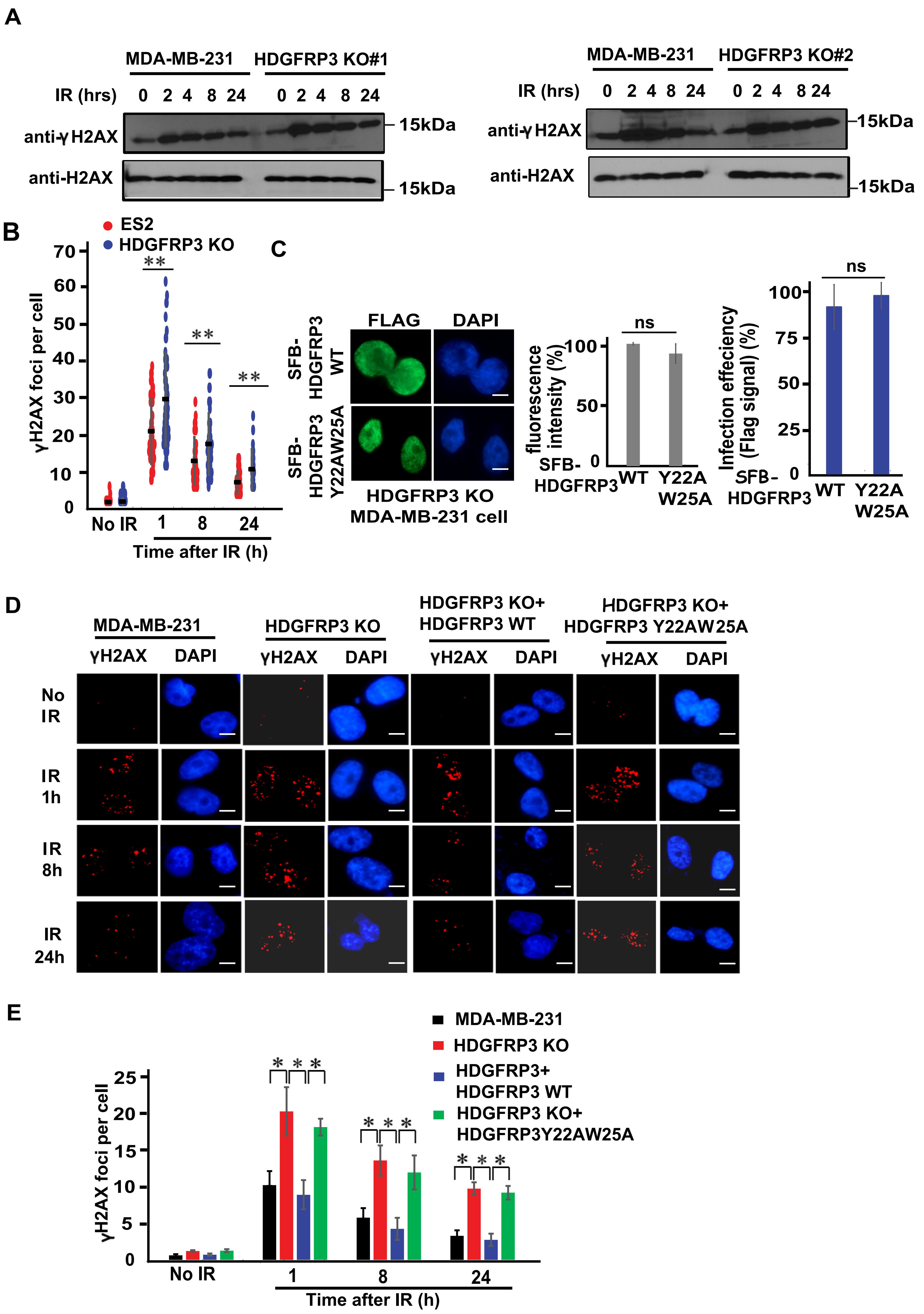

Zhang_Supplemental Figure S4

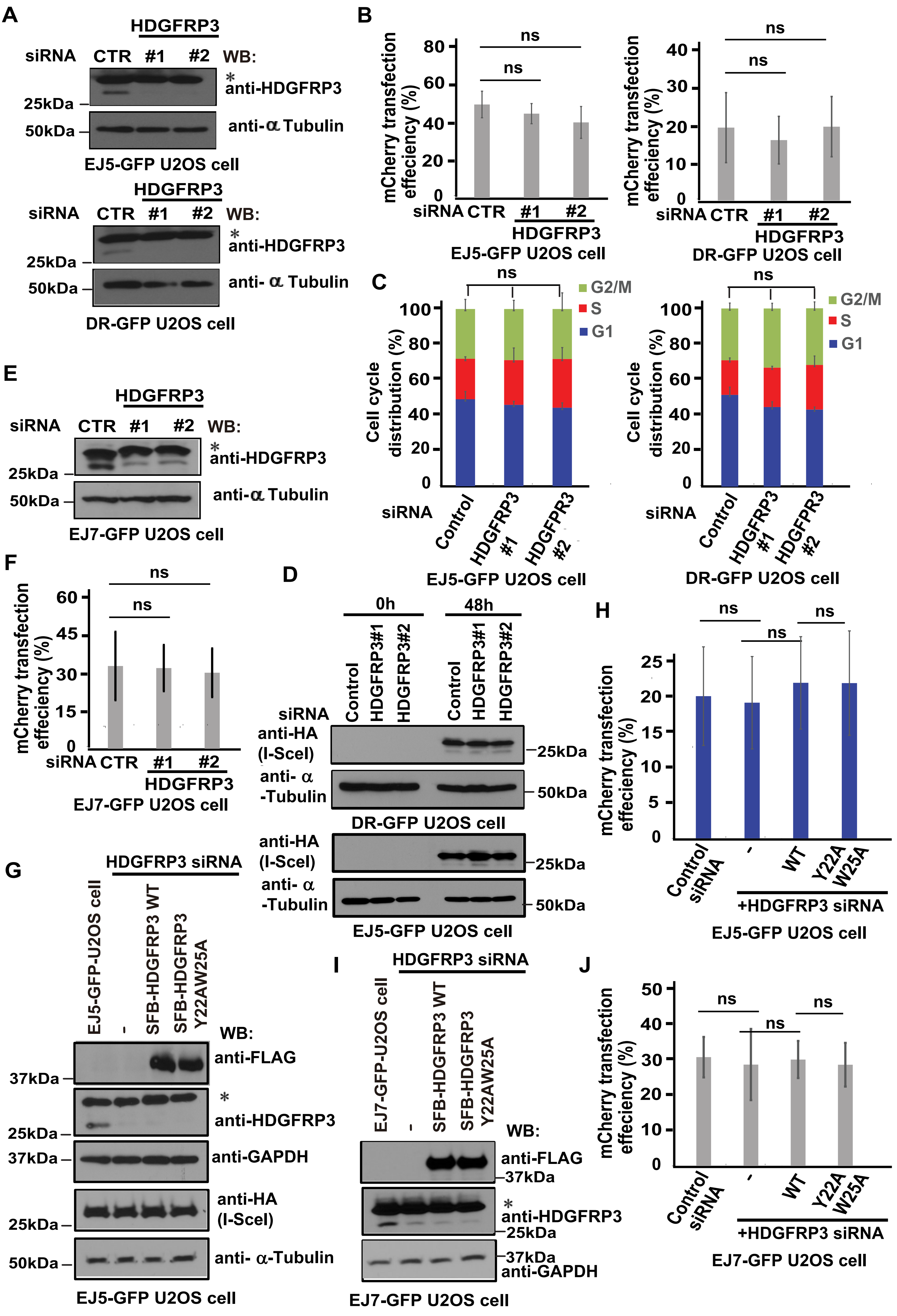

Zhang_Supplemenatl Figure S5

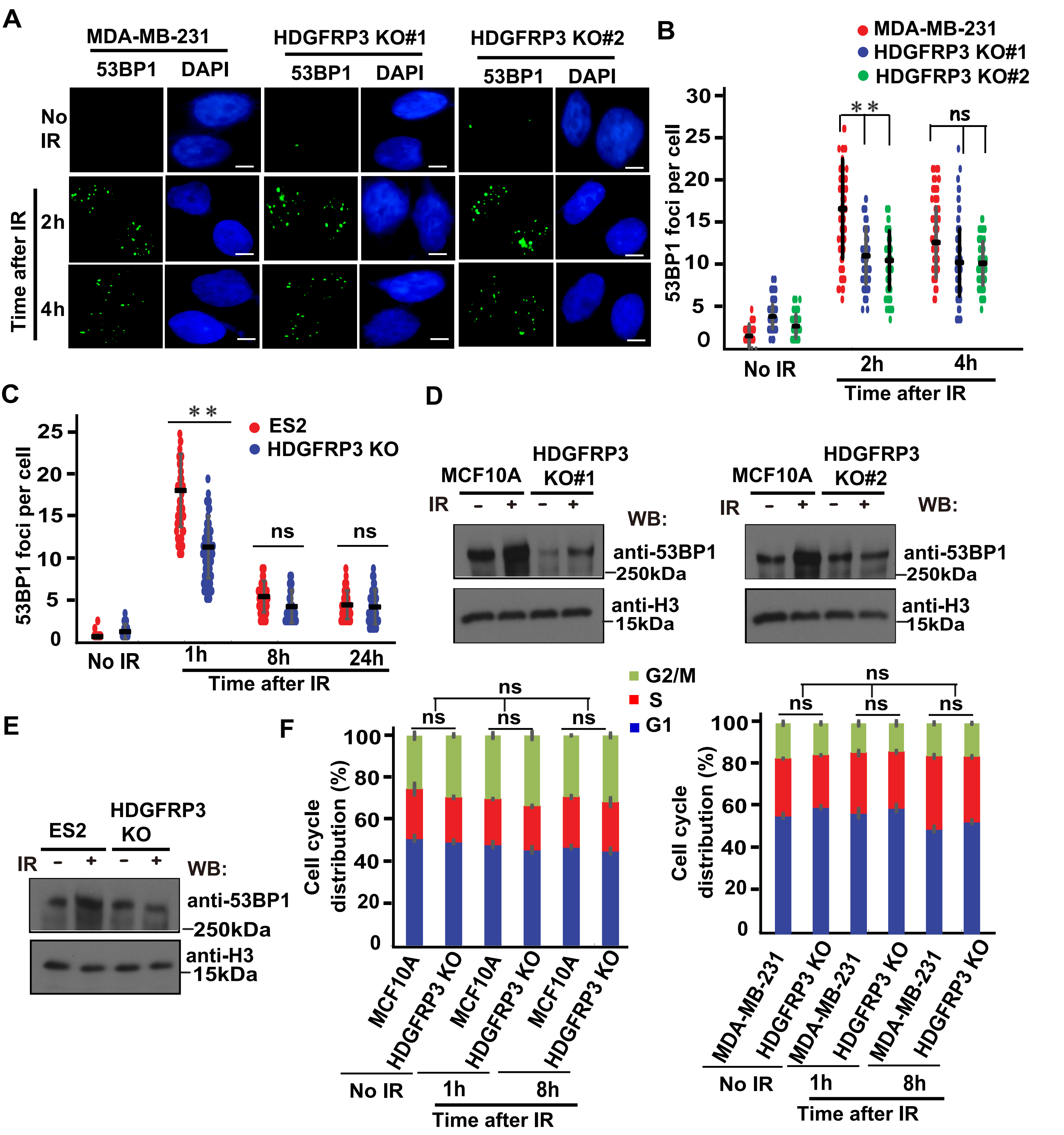

Zhang_Supplemental Figure S6

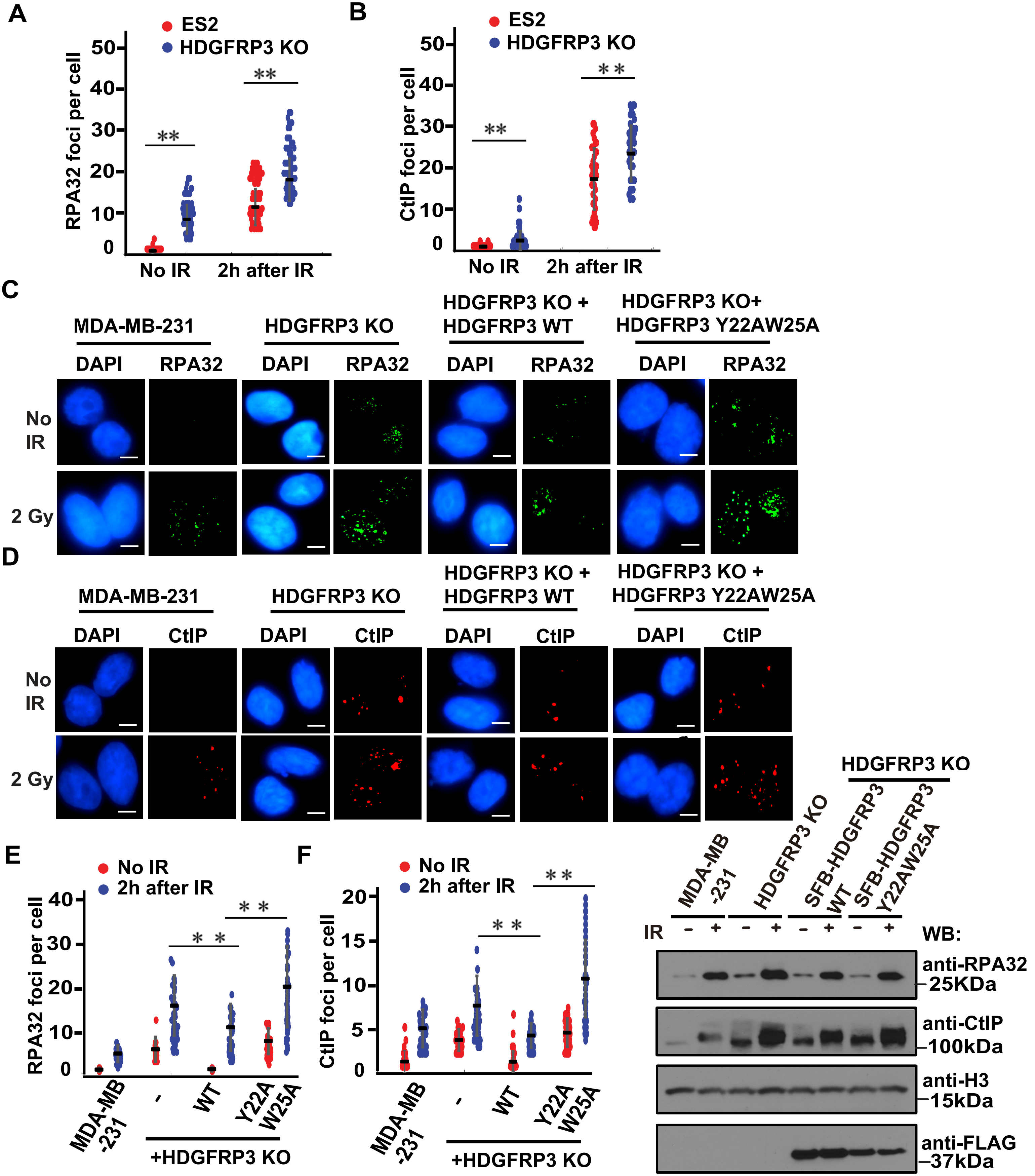

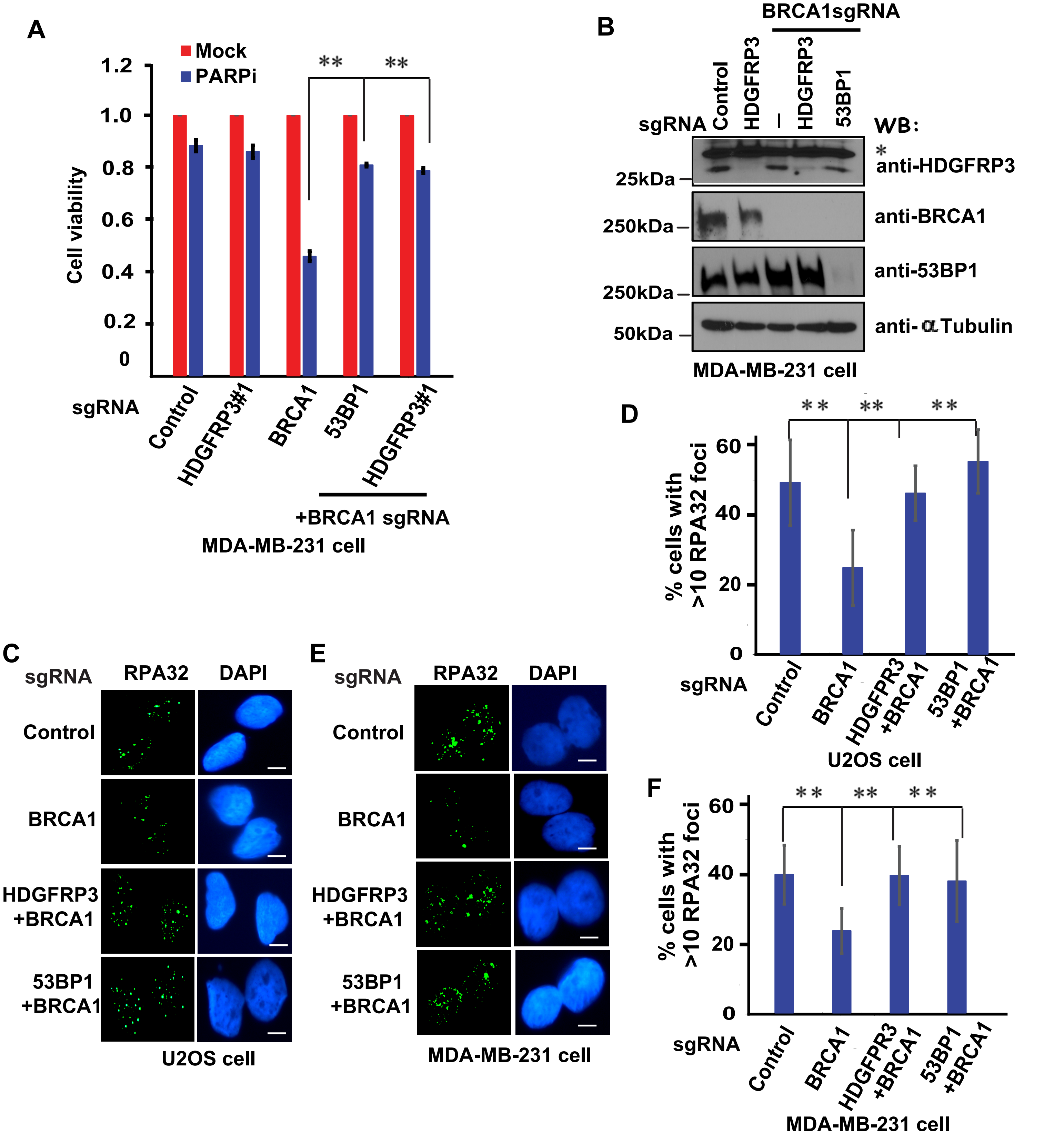
A
Mock
PARPi
1.2
1.0
0.8
0.6
0.4
0.2
0
Cell viability
** **
sgRNA
Control
HDGFRP3#1
BRCA1
53BP1
HDGFRP3#1
+BRCA1 sgRNA
MDA-MB-231 cell
B
BRCA1sgRNA
sgRNA
Control
HDGFRP3
–
HDGFRP3
53BP1
WB:
*
anti-HDGFRP3
25kDa
anti-BRCA1
250kDa
anti-53BP1
250kDa
anti-αTubulin
50kDa
MDA-MB-231 cell
C
sgRNA
RPA32
DAPI
Control
BRCA1
HDGFRP3
+BRCA1
53BP1
+BRCA1
U2OS cell
D
** ** **
% cells with
>10 RPA32 foci
60
40
20
0
sgRNA
Control
BRCA1
HDGFPR3
+BRCA1
53BP1
+BRCA1
U2OS cell
E
sgRNA
RPA32
DAPI
Control
BRCA1
HDGFRP3
+BRCA1
53BP1
+BRCA1
MDA-MB-231 cell
F
** ** **
% cells with
>10 RPA32 foci
60
40
20
0
sgRNA
Control
BRCA1
HDGFPR3
+BRCA1
53BP1
+BRCA1
MDA-MB-231 cell

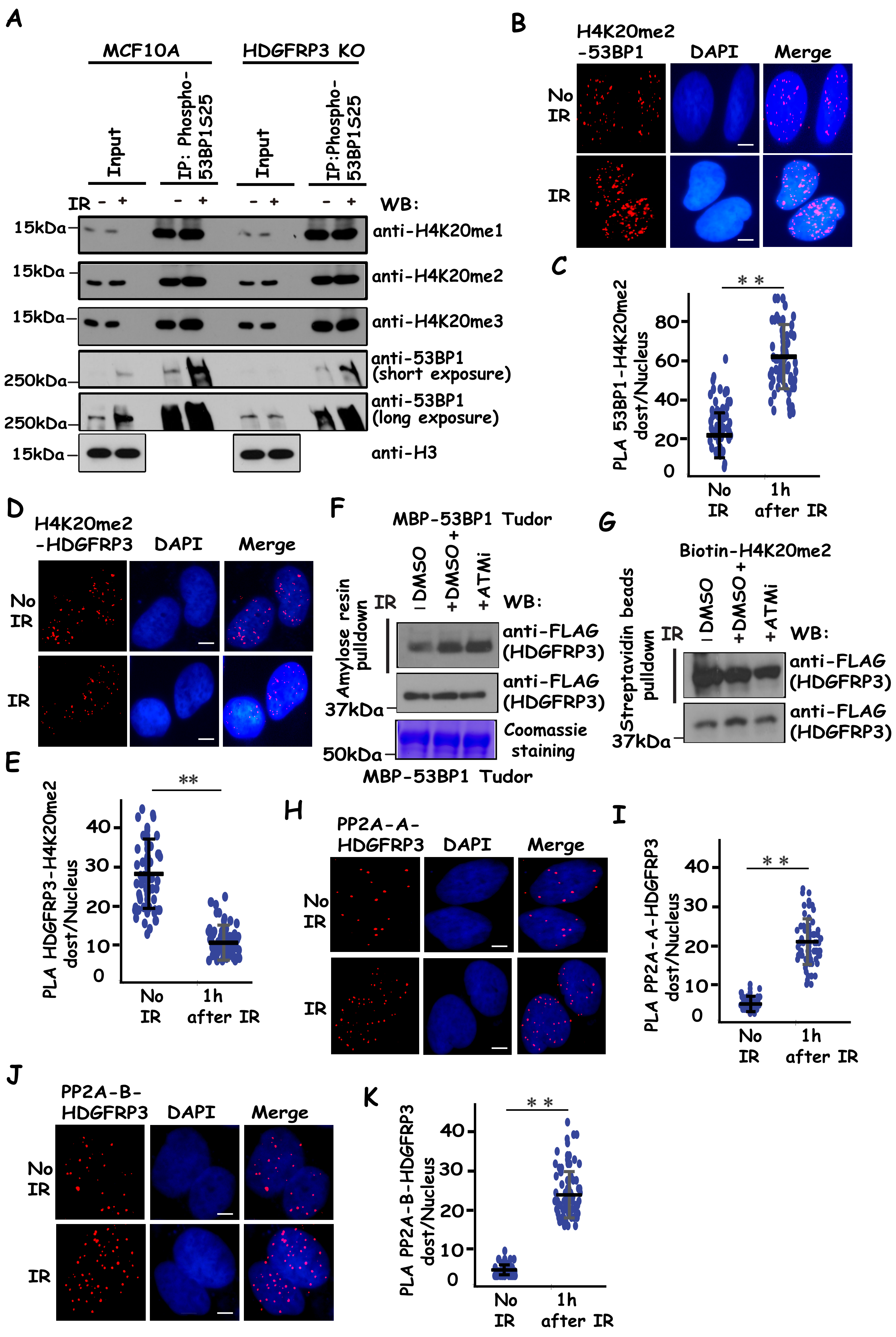
A
MCF10A
HDGFRP3 KO
Input
IP: Phospho-53BP1S25
Input
IP:Phospho-53BP1S25
IR - + - + - + - +
WB:
15kDa
anti-H4K20me1
15kDa
anti-H4K20me2
15kDa
anti-H4K20me3
250kDa
anti-53BP1 (short exposure)
250kDa
anti-53BP1 (long exposure)
15kDa
anti-H3
B
H4K20me2-53BP1
DAPI
Merge
No IR
IR
C
PLA 53BP1-H4K20me2 dost/Nucleus
**
80
60
40
20
0
No IR
1h after IR
D
H4K20me2-HDGFRP3
DAPI
Merge
No IR
IR
E
PLA HDGFRP3-H4K20me2 dost/Nucleus
**
40
30
20
10
0
No IR
1h after IR
F
MBP-53BP1 Tudor
Amylose resin pulldown
IR
- DMSO
+DMSO
+ATMi
WB:
anti-FLAG (HDGFRP3)
37kDa
anti-FLAG (HDGFRP3)
50kDa
Coomassie staining
MBP-53BP1 Tudor
G
Biotin-H4K20me2
Streptavidin beads pulldown
IR
- DMSO
+DMSO
+ATMi
WB:
anti-FLAG (HDGFRP3)
37kDa
anti-FLAG (HDGFRP3)
H
PP2A-A-HDGFRP3
DAPI
Merge
No IR
IR
I
PLA PP2A-A-HDGFRP3 dost/Nucleus
**
40
30
20
10
0
No IR
1h after IR
J
PP2A-B-HDGFRP3
DAPI
Merge
No IR
IR
K
PLA PP2A-B-HDGFRP3 dost/Nucleus
**
40
30
20
10
0
No IR
1h after IR

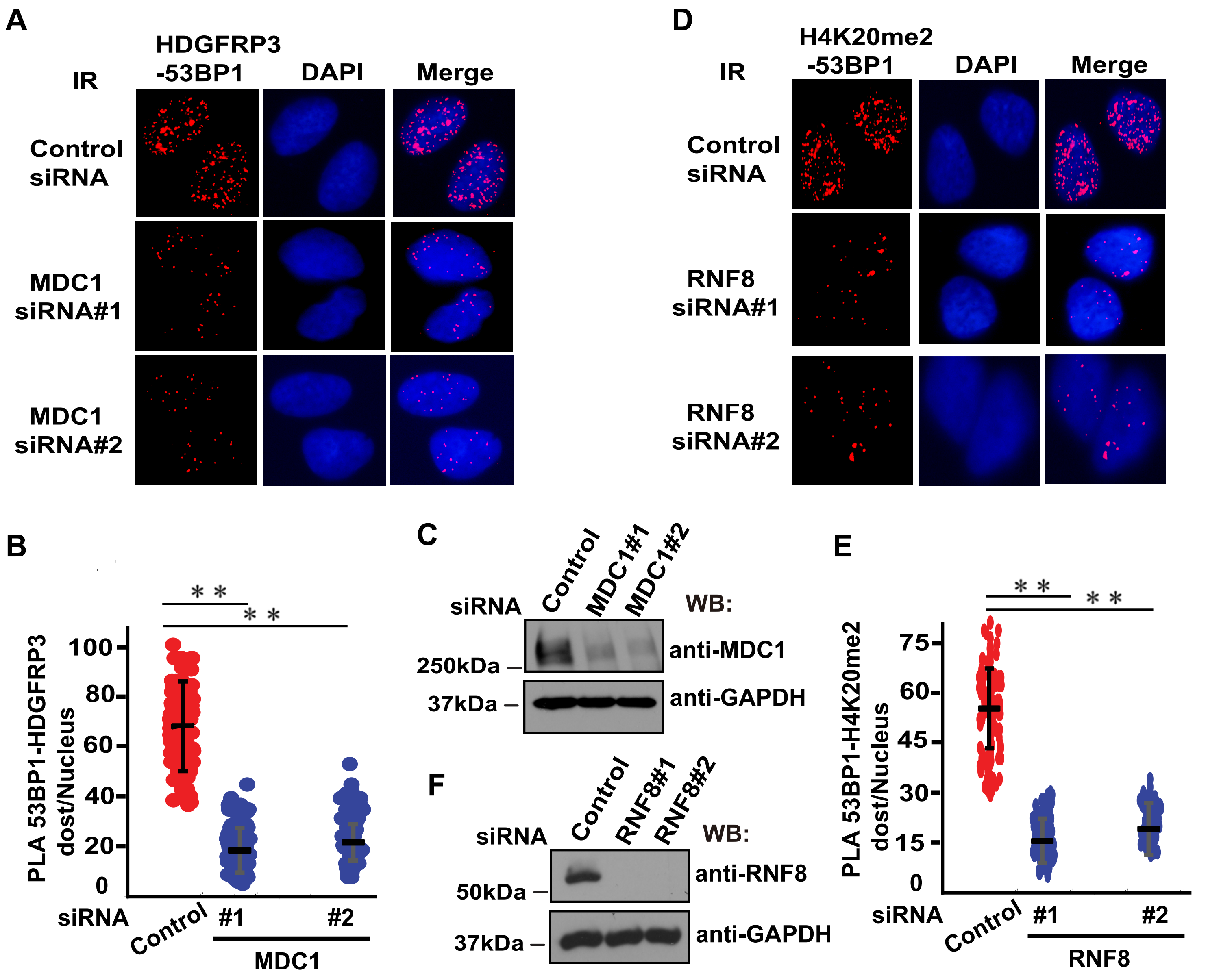
A
IR
HDGFRP3
-53BP1
DAPI
Merge
Control
siRNA
MDC1
siRNA#1
MDC1
siRNA#2
D
IR
H4K20me2
-53BP1
DAPI
Merge
Control
siRNA
RNF8
siRNA#1
RNF8
siRNA#2
B
PLA 53BP1-HDGFRP3
dost/Nucleus
100
80
60
40
20
0
**
**
siRNA
Control
#1
#2
MDC1
C
siRNA
Control
MDC1#1
MDC1#2
WB:
250kDa
anti-MDC1
37kDa
anti-GAPDH
F
siRNA
Control
RNF8#1
RNF8#2
WB:
50kDa
anti-RNF8
37kDa
anti-GAPDH
E
PLA 53BP1-H4K20me2
dost/Nucleus
75
60
45
30
15
0
**
**
siRNA
Control
#1
#2
RNF8

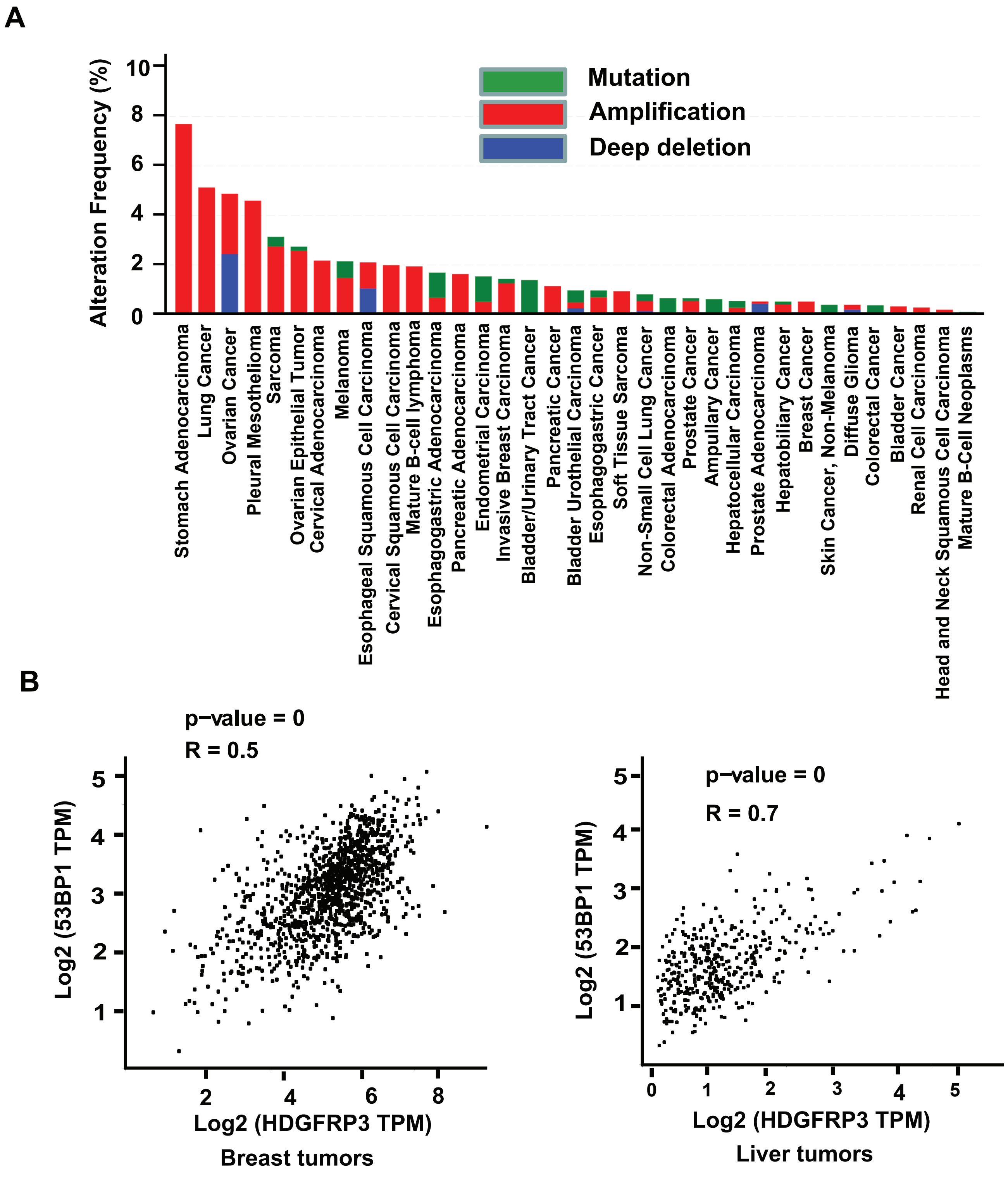
A
Alteration Frequency (%)
10
8
6
4
2
0
Mutation
Amplification
Deep deletion
Stomach Adenocarcinoma
Lung Cancer
Ovarian Cancer
Pleural Mesotheliloma
Sarcoma
Ovarian Epithelial Tumor
Cervical Adenocarcinoma
Melanoma
Esophageal Squamous Cell Carcinoma
Cervical Squamous Cell Carcinoma
Mature B-cell lymphoma
Esophagogastric Adenocarcinoma
Pancreatic Adenocarcinoma
Endometrial Carcinoma
Invasive Breast Carcinoma
Bladder/Urinary Tract Cancer
Pancreatic Cancer
Bladder Urothelial Carcinoma
Esophagogastric Cancer
Soft Tissue Sarcoma
Non-Small Cell Lung Cancer
Colorectal Adenocarcinoma
Prostate Cancer
Ampullary Cancer
Hepatocellular Carcinoma
Prostate Adenocarcinoma
Hepatobiliary Cancer
Breast Cancer
Skin Cancer, Non-Melanoma
Diffuse Glioma
Colorectal Cancer
Bladder Cancer
Renal Cell Carcinoma
Head and Neck Squamous Cell Carcinoma
Mature B-Cell Neoplasms
B
p−value = 0
R = 0.5
Log2 (53BP1 TPM)
Log2 (HDGFRP3 TPM)
Breast tumors
p−value = 0
R = 0.7
Log2 (53BP1 TPM)
Log2 (HDGFRP3 TPM)
Liver tumors

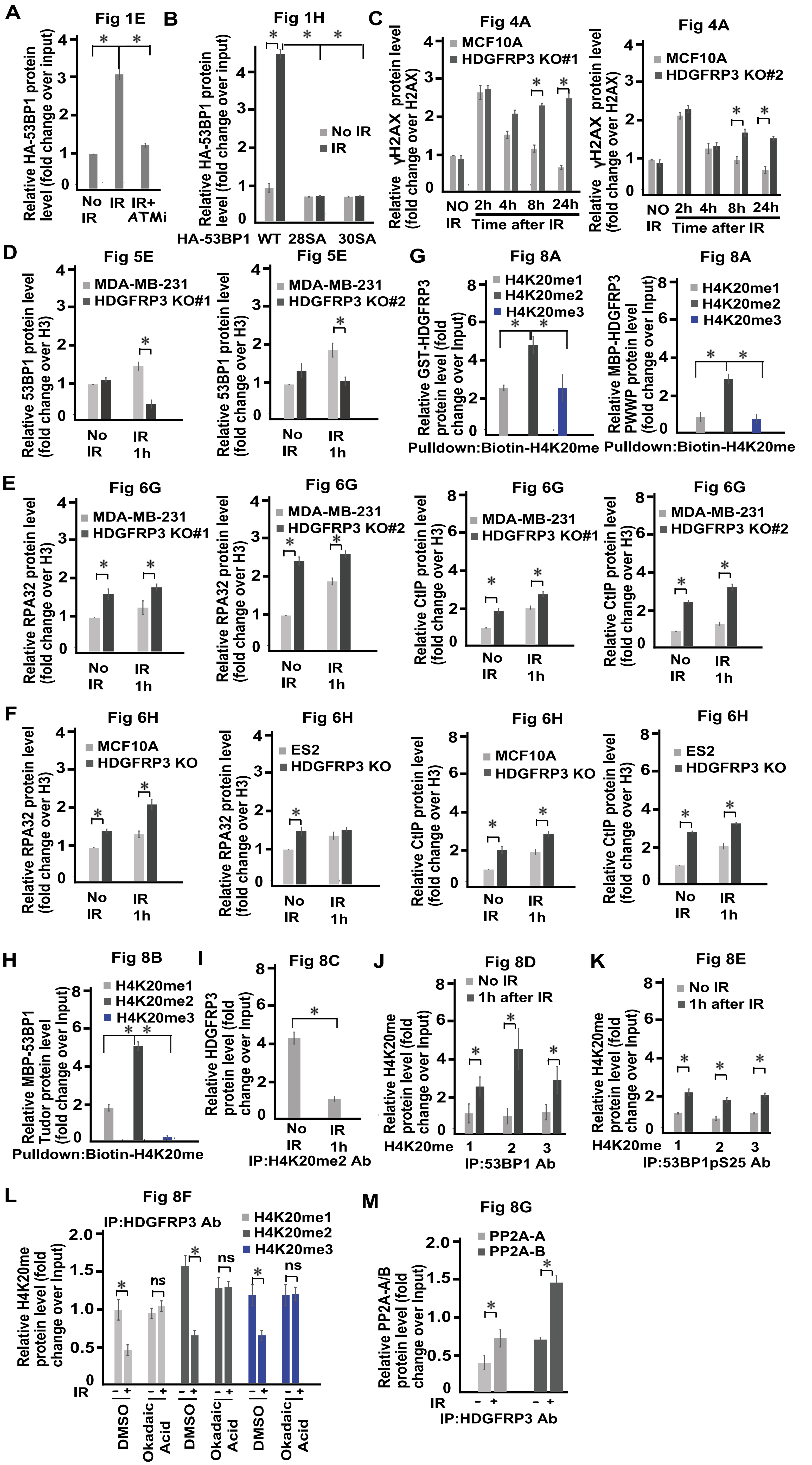
A
Fig 1E
Relative HA-53BP1 protein level (fold change over input)
No IR
IR
IR+ ATMi
B
Fig 1H
Relative HA-53BP1 protein level (fold change over input)
No IR
IR
HA-53BP1 WT 28SA 30SA
C
Fig 4A
Relative γH2AX protein level (fold change over H2AX)
MCF10A
HDGFRP3 KO#1
HDGFRP3 KO#2
NO IR
2h 4h 8h 24h
Time after IR
D
Fig 5E
Relative 53BP1 protein level (fold change over H3)
MDA-MB-231
HDGFRP3 KO#1
HDGFRP3 KO#2
No IR
IR 1h
G
Fig 8A
Relative GST-HDGFRP3 protein level (fold change over Input)
Relative MBP-HDGFRP3 PWWP protein level (fold change over Input)
H4K20me1
H4K20me2
H4K20me3
Pulldown:Biotin-H4K20me
E
Fig 6G
Relative RPA32 protein level (fold change over H3)
Relative CtIP protein level (fold change over H3)
MDA-MB-231
HDGFRP3 KO#1
HDGFRP3 KO#2
F
Fig 6H
MCF10A
ES2
HDGFRP3 KO
H
Fig 8B
Relative MBP-53BP1 Tudor protein level (fold change over Input)
Pulldown:Biotin-H4K20me
I
Fig 8C
Relative HDGFRP3 protein level (fold change over Input)
IP:H4K20me2 Ab
J
Fig 8D
Relative H4K20me protein level (fold change over Input)
No IR
1h after IR
H4K20me 1 2 3
IP:53BP1 Ab
K
Fig 8E
IP:53BP1pS25 Ab
L
Fig 8F
IP:HDGFRP3 Ab
H4K20me1
H4K20me2
H4K20me3
ns
IR
DMSO
Okadaic Acid
M
Fig 8G
Relative PP2A-A/B protein level (fold change over Input)
PP2A-A
PP2A-B
IP:HDGFRP3 Ab

Zhang_Supplemental Figure S12

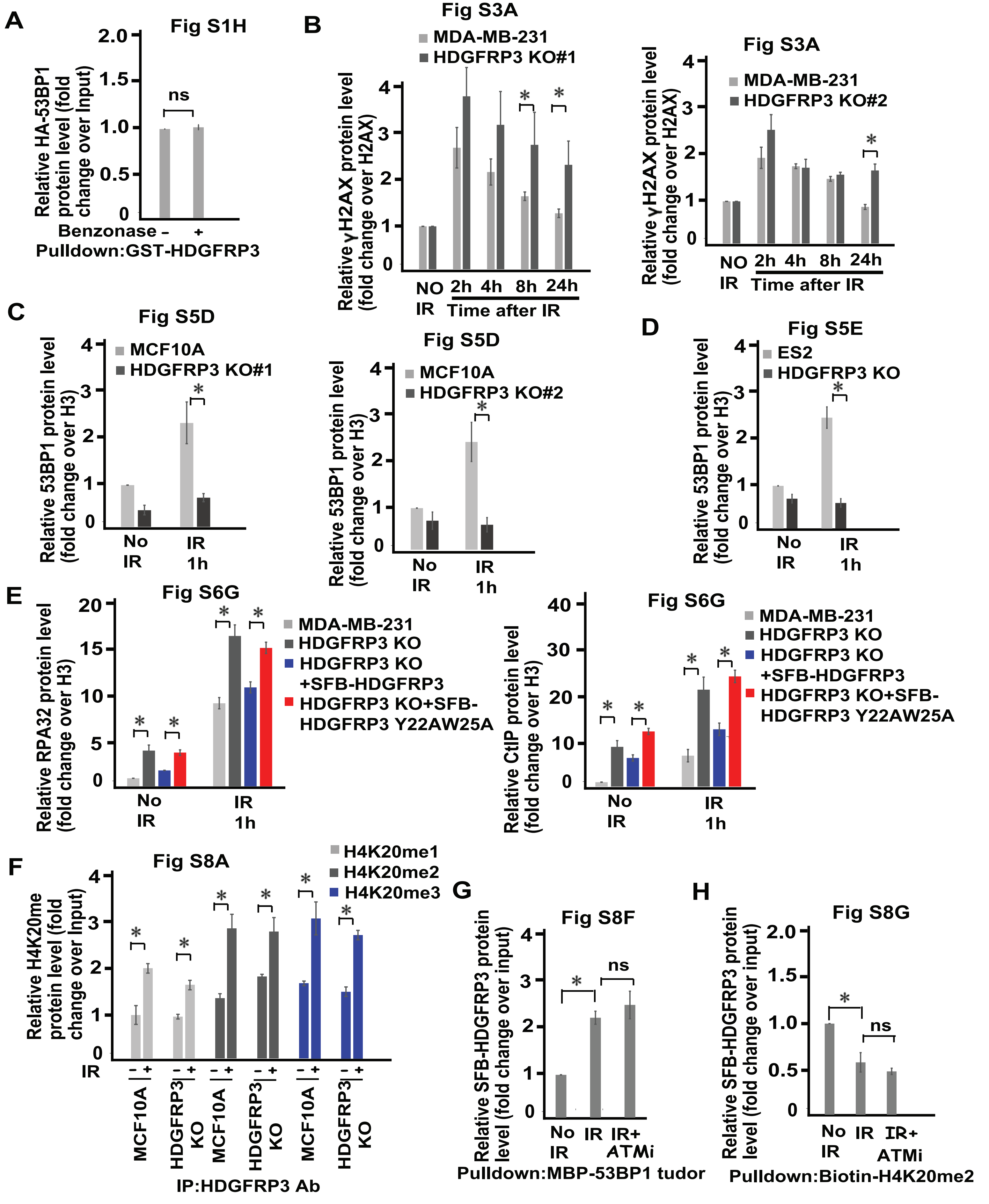

Supplement: gkad073_Supplemental_Files [file gkad073_supplemental_files.zip › Supplemental Figures.pdf]
